# Nutritional quality of food as represented by the FSAm-NPS nutrient profiling system underlying the Nutri-Score label and cancer risk in Europe: Results from the EPIC prospective cohort study

**DOI:** 10.1371/journal.pmed.1002651

**Published:** 2018-09-18

**Authors:** Mélanie Deschasaux, Inge Huybrechts, Neil Murphy, Chantal Julia, Serge Hercberg, Bernard Srour, Emmanuelle Kesse-Guyot, Paule Latino-Martel, Carine Biessy, Corinne Casagrande, Mazda Jenab, Heather Ward, Elisabete Weiderpass, Christina C. Dahm, Kim Overvad, Cecilie Kyrø, Anja Olsen, Aurélie Affret, Marie-Christine Boutron-Ruault, Yahya Mahamat-Saleh, Rudolf Kaaks, Tilman Kühn, Heiner Boeing, Lukas Schwingshackl, Christina Bamia, Eleni Peppa, Antonia Trichopoulou, Giovanna Masala, Vittorio Krogh, Salvatore Panico, Rosario Tumino, Carlotta Sacerdote, Bas Bueno-de-Mesquita, Petra H. Peeters, Anette Hjartåker, Charlotta Rylander, Guri Skeie, J. Ramón Quirós, Paula Jakszyn, Elena Salamanca-Fernández, José María Huerta, Eva Ardanaz, Pilar Amiano, Ulrika Ericson, Emily Sonestedt, Ena Huseinovic, Ingegerd Johansson, Kay-Tee Khaw, Nick Wareham, Kathryn E. Bradbury, Aurora Perez-Cornago, Konstantinos K. Tsilidis, Pietro Ferrari, Elio Riboli, Marc J. Gunter, Mathilde Touvier

**Affiliations:** 1 Nutritional Epidemiology Research Team (EREN), Sorbonne Paris Cité Epidemiology and Statistics Research Centre (CRESS), Inserm U1153, Inra U1125, Cnam, COMUE Sorbonne Paris Cité, Paris 13 University, Bobigny, France; 2 Nutrition and Metabolism Section, International Agency for Research on Cancer, World Health Organization, Lyon, France; 3 Department of Public Health, Avicenne Hospital (AP-HP), Bobigny, France; 4 Faculty of Medicine, School of Public Health, Imperial College London, London, United Kingdom; 5 Department of Community Medicine, Faculty of Health Sciences, UiT The Arctic University of Norway, Tromsø, Norway; 6 Department of Research, Cancer Registry of Norway, Institute of Population-Based Cancer Research, Oslo, Norway; 7 Department of Medical Epidemiology and Biostatistics, Karolinska Institutet, Stockholm, Sweden; 8 Genetic Epidemiology Group, Folkhälsan Research Centre and Faculty of Medicine, University of Helsinki, Helsinki, Finland; 9 Aarhus University, Department of Public Health, Section for Epidemiology, Aarhus C, Denmark; 10 Danish Cancer Society Research Center, Copenhagen, Denmark; 11 CESP, INSERM U1018, Univ. Paris-Sud, UVSQ, Université Paris-Saclay, Paris, France; 12 Gustave Roussy, Villejuif, France; 13 Division of Cancer Epidemiology, German Cancer Research Center (DKFZ), Heidelberg, Germany; 14 Department of Epidemiology, German Institute of Human Nutrition (DIfE), Potsdam-Rehbrücke, Germany; 15 Hellenic Health Foundation, Athens, Greece; 16 WHO Collaborating Center for Nutrition and Health, Unit of Nutritional Epidemiology and Nutrition in Public Health, Dept. of Hygiene, Epidemiology and Medical Statistics, School of Medicine, National and Kapodistrian University of Athens, Athens, Greece; 17 Cancer Risk Factors and Life-Style Epidemiology Unit, Cancer Research and Prevention Institute–ISPO, Florence, Italy; 18 Epidemiology and Prevention Unit, Fondazione IRCCS Istituto Nazionale dei Tumori, Milano, Italy; 19 Dipartimento di Medicina Clinica e Chirurgia, Federico II University, Naples, Italy; 20 Cancer registry and histopathology unit, "CIVIC-M.P. AREZZO" Hospital, ASP Ragusa, Italy; 21 Unit of Cancer Epidemiology, Città della Salute e della Scienza University-Hospital and Center for Cancer Prevention (CPO), Turin, Italy; 22 Department for Determinants of Chronic Diseases (DCD), National Institute for Public Health and the Environment (RIVM), Bilthoven, The Netherlands; 23 Department of Gastroenterology and Hepatology, University Medical Centre, Utrecht, The Netherlands; 24 Department of Epidemiology and Biostatistics, The School of Public Health, Imperial College London, London, United Kingdom; 25 Department of Social & Preventive Medicine, Faculty of Medicine, University of Malaya, Kuala Lumpur, Malaysia; 26 Department of Epidemiology, Julius Center for Health Sciences and Primary Care, University Medical Center Utrecht, Utrecht University, Utrecht, The Netherlands; 27 Department of Nutrition, Institute of Basic Medical Sciences, University of Oslo, Oslo, Norway; 28 Public Health Directorate, Asturias, Spain; 29 Unit of Nutrition, Environment and Cancer, Cancer Epidemiology Research Programme, Catalan Institute of Oncology, L´Hospitallet de Llobregat, Barcelona, Spain; 30 Facultat de Ciències de la Salut Blanquerna, Universitat Ramón Llull, Barcelona, Spain; 31 Escuela Andaluza de Salud Pública. Instituto de Investigación Biosanitaria ibs.GRANADA, Hospitales Universitarios de Granada/Universidad de Granada, Granada, Spain; 32 CIBER de Epidemiología y Salud Pública (CIBERESP), Madrid, Spain; 33 Department of Epidemiology, Murcia Regional Health Council, IMIB-Arrixaca, Murcia, Spain; 34 Navarra Public Health Institute, Pamplona, Spain; 35 IdiSNA, Navarra Institute for Health Research, Pamplona, Spain; 36 Public Health Department of Gipuzkoa, Basque Government, San Sebastian, Spain; 37 Diabetes and Cardiovascular disease, Genetic Epidemiology, Department of Clinical Sciences, Lund University, Malmö, Sweden; 38 Nutritional Epidemiology, Department of Clinical Sciences Malmö, Lund University, Malmö, Sweden; 39 Department of Internal Medicine and Clinical Nutrition, The Sahlgrenska Academy, University of Gothenburg, Gothenburg, Sweden; 40 Department of Odontology, Umea University, Umea, Sweden; 41 University of Cambridge, School of Clinical Medicine, Addenbrooke’s Hospital, Cambridge, United Kingdom; 42 MRC Epidemiology Unit, Institute of Metabolic Science, University of Cambridge, Cambridge, United Kingdom; 43 Cancer Epidemiology Unit, Nuffield Department of Population Health, University of Oxford, Oxford, United Kingdom; 44 Department of Hygiene and Epidemiology, University of Ioannina School of Medicine, Ioannina, Greece; Vanderbilt University School of Medicine, UNITED STATES

## Abstract

**Background:**

Helping consumers make healthier food choices is a key issue for the prevention of cancer and other diseases. In many countries, political authorities are considering the implementation of a simplified labelling system to reflect the nutritional quality of food products. The Nutri-Score, a five-colour nutrition label, is derived from the Nutrient Profiling System of the British Food Standards Agency (modified version) (FSAm-NPS). How the consumption of foods with high/low FSAm-NPS relates to cancer risk has been studied in national/regional cohorts but has not been characterized in diverse European populations.

**Methods and findings:**

This prospective analysis included 471,495 adults from the European Prospective Investigation into Cancer and Nutrition (EPIC, 1992–2014, median follow-up: 15.3 y), among whom there were 49,794 incident cancer cases (main locations: breast, *n* = 12,063; prostate, *n* = 6,745; colon-rectum, *n* = 5,806). Usual food intakes were assessed with standardized country-specific diet assessment methods. The FSAm-NPS was calculated for each food/beverage using their 100-g content in energy, sugar, saturated fatty acid, sodium, fibres, proteins, and fruits/vegetables/legumes/nuts. The FSAm-NPS scores of all food items usually consumed by a participant were averaged to obtain the individual FSAm-NPS Dietary Index (DI) scores. Multi-adjusted Cox proportional hazards models were computed. A higher FSAm-NPS DI score, reflecting a lower nutritional quality of the food consumed, was associated with a higher risk of total cancer (HR_Q5_ versus _Q1_ = 1.07; 95% CI 1.03–1.10, *P*-trend < 0.001). Absolute cancer rates in those with high and low (quintiles 5 and 1) FSAm-NPS DI scores were 81.4 and 69.5 cases/10,000 person-years, respectively. Higher FSAm-NPS DI scores were specifically associated with higher risks of cancers of the colon-rectum, upper aerodigestive tract and stomach, lung for men, and liver and postmenopausal breast for women (all *P* < 0.05). The main study limitation is that it was based on an observational cohort using self-reported dietary data obtained through a single baseline food frequency questionnaire; thus, exposure misclassification and residual confounding cannot be ruled out.

**Conclusions:**

In this large multinational European cohort, the consumption of food products with a higher FSAm-NPS score (lower nutritional quality) was associated with a higher risk of cancer. This supports the relevance of the FSAm-NPS as underlying nutrient profiling system for front-of-pack nutrition labels, as well as for other public health nutritional measures.

## Abstract 2

In a research article, Mélanie Deschasaux and colleagues study associations between an indicator of the nutritional quality of individuals' food consumption and cancer risk.

## Author summary

**Why was this study done?:**

- Helping consumers make healthier food choices is a key challenge for the prevention of cancer and other chronic diseases, which is why in many countries, political authorities are considering the implementation of a simplified labelling system to reflect the nutritional quality of food products.
- The Nutri-Score, a five-colour nutrition label based on the Nutrient Profiling System of the British Food Standards Agency (modified version) (FSAm-NPS) score (calculated for each food/beverage using its 100-g content in energy, sugar, saturated fatty acids, sodium, fibres, proteins, and fruits/vegetables/legumes/nuts) has been selected by French authorities but remains optional per European labelling regulations.
- So far, scientific evidence regarding the relevance of the Nutri-Score (and the underlying FSAm-NPS score) has been obtained at national/regional level, thus expanding investigations to the European level is of importance.

**What did the researchers do and find?:**

- This study is part of a comprehensive assessment of the FSAm-NPS validity as underlying nutrient profiling system for front-of-pack nutrition labels (as well as other public health nutritional measures) in Europe.
- Here, we conducted a prospective analysis of the association between the FSAm-NPS score of the food consumed (reflecting their nutritional quality) and cancer risk in the large and diverse European population that constitutes the European Prospective Investigation into Cancer and Nutrition (EPIC) cohort, including 471,495 adults from 10 European countries with 49,794 newly diagnosed cancer cases.
- The consumption of foods with higher FSAm-NPS scores, reflecting a lower nutritional quality, was associated with an increased risk of developing cancer (overall and several specific cancer sites).

**What do these findings mean?:**

- These findings add support to the relevance of using the FSAm-NPS to grade the nutritional quality of food products as a basis for prevention strategies for cancer and other chronic diseases.
- These findings will play a role in communications about the merits of the Nutri-Score to consumers, healthcare professionals and economic operators, in the context of the ongoing European/international debate on nutritional labelling.

## Introduction

About a third of the most common cancers in Western countries are estimated to be preventable through appropriate nutritional behaviours (World Cancer Research Fund [WCRF]/American Institute for Cancer Research [AICR]) [1]. If nutrition can be modified at the individual level and therefore targeted by public health policies, informing the general population to make healthy, evidence-based nutritional decisions remains an important challenge. Among the promising strategies proposed to promote a healthier dietary environment [2,3], simplified front-of-pack nutrition labels, providing summarized, easy-to-use information on the nutritional quality of food products, have the potential to help consumers make healthier food choices and to encourage the food industry to improve the nutritional quality of the food supply [4,5]. The Nutri-Score five-colour labelling system (see S1 Fig) [3] uses a modified version of the British Food Standards Agency Nutrient Profiling System (original version) (FSA-NPS) [6,7], considered a promising nutrient profiling system for use in a broad international context [6,8], to categorize food products into 5 colours reflecting their nutritional quality (see examples in S1 Text). The FSA-NPS was built in a perspective of prevention of a large range of chronic diseases. It allocates a score to a given food/beverage from its content per 100 g of energy, saturated fatty acids, sugar, sodium, dietary fibres, proteins, and fruit/vegetables/legumes/nuts. It was initially developed and validated in the United Kingdom, where it has been used for advertising regulation (Ofcom) [6,7,9] and was transposed in France (FSAm-NPS) [10–12].

Several studies support the scientific relevance and the potential public health impact of the use of the FSAm-NPS as a basis for public health nutrition policies [13–21] (reviewed in [22]). In particular, studies performed in the SU.VI.MAX and NutriNet-Santé cohorts have shown that a diet composed of food products with better FSAm-NPS scores (summarized with the FSAm-NPS Dietary Index [DI] [23,24]) would lead to more favourable health outcomes as regards weight gain [25], metabolic syndrome [26], cardiovascular diseases [27,28], and cancer incidence (total and breast) [29,30]. These results were promising albeit restricted to French populations and based on a relatively limited number of cases (especially to perform robust analyses by cancer sites).

In 2017, the Nutri-Score was selected by the French Ministry of Health as the official front-of-pack nutrition label to be implemented in France [31,32], an initiative officially commended by WHO Europe [33]. However, to comply with the European Union (EU) labelling regulations, appending the Nutri-Score on food products remains optional and therefore relies on voluntary uptake by food manufacturers. In 2018, a review of existing labelling schemes at the EU level is anticipated, and discussions regarding the possible implementation of a unique nutritional labelling system for all EU countries are expected to follow. Similar discussions are also ongoing in North and South America, Canada, and Australia. Scientific evidence regarding the relevance of this label (and the underlying FSAm-NPS score) at an international level is therefore of importance.

This study is part of a comprehensive assessment of the validity of the FSAm-NPS as underlying nutrient profiling system for front-of-pack nutrition labels as well as other public health nutritional measures in Europe. Specifically, it aimed at investigating the association between the FSAm-NPS DI and cancer risk in the large and diverse European population that constitutes the European Prospective Investigation into Cancer and Nutrition (EPIC) cohort.

## Methods

### Study population: The EPIC cohort

EPIC (http://epic.iarc.fr/) is a multicentre prospective cohort study investigating metabolic, dietary, lifestyle, and environmental factors in relation to cancer and other chronic diseases. Between 1992 and 2000, more than 500,000 volunteers (25–70 years old) were recruited from 10 European countries (23 administrative centres): Denmark, France, Germany, Greece, Italy, the Netherlands, Norway, Spain, Sweden, and the UK. All participants gave written informed consent. The study was approved by the local ethics committees and by the Internal Review Board of the International Agency for Research on Cancer. Details of the study design, recruitment, and data collection have been previously published [34–36].

Of the 521,324 participants enrolled, 471,495 were included in the analyses (see flowchart in S2 Fig for exclusion details). In particular, from the 54,459 eligible invasive cancer cases, we excluded those diagnosed within the first 2 years of follow-up (*n* = 4,665) to allow sufficient delay between baseline dietary assessment and cancer diagnosis, thereby limiting reverse causality.

### Baseline data collection

An extended and standardized phenotypic characterisation was performed for each participant upon enrolment. Questionnaires were used to collect sociodemographic information, educational level (collected and standardized for the whole cohort), personal and familial history of diseases, lifestyle (e.g., smoking, alcohol use, physical activity), and menstrual and reproductive history for women. Anthropometric measurements (e.g., height, weight, waist, and hip circumferences) were performed in all centres (except France, Oxford, and Norway: self-reported data).

### Dietary intake assessment

Usual dietary intake was assessed for each individual at recruitment using country-specific and validated dietary questionnaires developed to capture the geographical specificity of an individual’s diet. The type of dietary questionnaire used differed according to study centres and included: self- or interviewer-administered semiquantitative food frequency questionnaires (FFQs) with an estimation of individual average portions or with the same standard portion assigned to all subjects or diet history questionnaires combining an FFQ and 7-day dietary records [36]. The EPIC food composition database comprises more than 10,000 food and beverage items reflecting the specificities of each country [37].

### FSAm-NPS DI computation

As described previously [7,10,12], the FSAm-NPS score is a modified version of the original FSA-NPS, with adaptations in the allocation of points for beverages, cheese, and added fats following recommendations from the French High Council for Public Health (HCSP) to ensure a high consistency of the FSAm-NPS score with nutritional recommendations, for labelling purposes [12].

The FSAm-NPS score was calculated for all foods and beverages in the EPIC food composition database as follows: points (0–10) are allocated for the content per 100 g in total sugars (g), saturated fatty acids (g), sodium (mg), and energy (kJ) (i.e., nutrients that should be consumed in limited amounts) and can be balanced by opposite points (0–5) allocated for dietary fibres (g), proteins (g), and fruits/vegetables/legumes/nuts (percent) (i.e., nutrients/components that should be promoted). The grids for point attribution are displayed in S1 Text (general rule and specific grids: sugars, energy, and fruits/vegetables/legumes/nuts for beverages, saturated fatty acids for added fats). The percentage of fruits/vegetables/legumes/nuts was derived using standard recipes. The FSAm-NPS score for each food/beverage is based on a unique discrete continuous scale ranging theoretically from −15 (most healthy) to +40 (least healthy). The universality of the FSAm-NPS components allows a computation for all existing foods/beverages, no matter the cultural diet structure in which they are included.

An individual consumes many different foods of contrasted nutritional quality, which synergistically influence his/her disease risk. When studying the association between food intakes and chronic diseases, all food items consumed have to be considered (and therefore all associated FSAm-NPS scores) and not just one single food. Therefore, in a second step, the FSAm-NPS DI was computed at the individual level as an energy-weighted mean of the FSAm-NPS scores of all foods and beverages consumed using the following equation [23] (FS_i_: score of food/beverage i, E_i_: energy intake from food/beverage i, n: number of food/beverage consumed):

$$\mathrm{FSAm}‑\mathrm{NPS}\;\mathrm{DI}=\frac{\sum_{i=1}^{n}{(FS}_{i}E_{i})}{\sum_{i=1}^{n}E_{i}}$$

Higher FSAm-NPS DI therefore reflects lower nutritional quality in foods consumed.

More details on FSAm-NPS and FSAm-NPS DI calculations can be found in S1 Text.

### Follow-up for cancer incidence and vital status

Incident cancer cases were identified through several methods, including record linkage with population-based cancer registries, health insurance records, pathology registries, and active follow-up of study subjects. Data on vital status were obtained from mortality registries, in combination with data collected through active follow-up. The end of follow-up/closure dates of the study period varied between 2009 and 2014 depending on the countries.

First primary invasive cancers were considered as cases in this study. Main cancer cases were coded according to the International Classification of Diseases for Oncology (ICD-O) [38] as follows: colorectal cancer (C18, C199, C209), bladder cancer (C67), kidney cancer (C649), upper aerodigestive tract cancers (oral cavity: C019, C02, C03, C04, C050, C06; oropharynx: C09, C10; hypopharynx: C13, C14; larynx: C32; esophagus: C15), lung cancer (C34), stomach cancer (C16), pancreas cancer (C25), liver cancer (C220), breast cancer (C50), endometrial cancer (C54), cervical cancer (C53), ovary cancer (C569), prostate cancer (C61).

### Statistical analyses

All statistical analyses were pre-planned and followed the plan detailed in the project protocol that was submitted for funding application (S2 Text). Associations between the FSAm-NPS DI (continuous variable and sex-specific quintiles) and cancer risk overall and for specific cancer locations were characterized (hazard ratio [HR] and 95% CI) using multivariable Cox proportional hazards models with age as the primary time variable. We confirmed that the assumptions of proportionality were satisfied through examination of the log–log (survival) versus log–time plots. Tests for linear trends were performed with an ordinal coding of FSAm-NPS DI quintiles (1, 2, 3, 4, 5). Participants contributed person-time to the model until their date of cancer diagnosis, their date of death, their date of emigration/loss to follow-up, or end-of-follow-up, whichever occurred first. Analysis by censoring the competing death event is the most appropriate way for HR estimation in evaluating exposure–disease associations [39,40]. For analyses of specific cancer sites, participants who reported a cancer other than the one under study were included and censored at the date of diagnosis (except basal cell skin carcinoma, which was not considered as cancer).

Analyses were performed for sexes combined and by sex. Models were stratified by age at recruitment (1-y intervals) and study centre [34] (‘strata’ option in proc phreg, SAS) and multivariable adjusted for other known risk factors for cancer: sex, body mass index (BMI), height, educational level, physical activity, smoking status and intensity, alcohol intake at recruitment, total energy intake, family history of breast and colorectal cancer, and, for women (subgroup analyses), menopausal status at baseline and whether they ever used hormonal treatment for menopause or oral contraception. For women-specific cancer locations (cancers of the reproductive system), models were further adjusted for age at menarche, age at first full-term pregnancy, age at menopause, and an interaction term between BMI and menopausal status. For these cancers, models were computed by menopausal status (pre-menopause/post-menopause): women contributed person-time to the ‘pre-menopause model’ until their age of menopause and to the ‘post-menopause model’ from their age of menopause. Detailed information about covariate categorization can be found in the table footnotes. Age at menopause was collected at baseline for postmenopausal women. If missing or if women were pre- or perimenopausal at baseline, then age at menopause was set at 55 years [41]. For analyses on cancers of the endometrium, cervix, and ovaries, we excluded women who declared a surgical menopause at baseline. When data on categorical covariates were missing, a ‘missing class’ was introduced in the model. If missing, height and weight were imputed with centre-, age-, and gender-specific average values. Sensitivity analyses were also performed using a ‘complete cases’ approach, excluding participants with missing data on covariates.

BMI was considered as a confounding factor in the analyses and thus was adjusted for in the models. However, BMI could also be considered as a potential intermediate factor, which was tested in a sensitivity analysis excluding BMI.

Unadjusted absolute rates were calculated as the number of cases per 10,000 person-years in the highest and the lowest quintiles, respectively, of the FSAm-NPS DI score.

All tests were two sided, and *P* < 0.05 was considered statistically significant. SAS version 9.4 (SAS Institute) was used for the analyses.

## Results

After a median follow-up time of 15.3 y (between 1992–2000 and 2009–2014), 49,794 incident invasive cancer cases were recorded (cancer incidence by country is shown in S1 Table). The most common cancers were breast (*n* = 12,063), prostate (*n* = 6,745), colon-rectum (*n* = 5,806), and lung (*n* = 3,654).

Participants with a higher FSAm-NPS DI score, reflecting a diet of lower nutritional quality, were consistently more likely to have unhealthy dietary intakes, e.g., higher intakes of alcohol, energy and red and processed meat, lower intakes of dietary fibres, vegetables, fruit, fish, and lean meat (Table 1). Participants from France, Germany, the UK (Cambridge centre), and Sweden were more likely to score higher on the FSAm-NPS DI (i.e., to consume food products of lower nutritional quality) and thus to be classified in the 5th quintile, whereas participants from Greece, Italy, Spain, Norway, and the UK (Oxford centre, mainly ‘health-conscious’ participants, including a high proportion of vegetarians) were more likely to have lower scores. Participants from Denmark and the Netherlands were more likely to have middle-range scores falling within the 2nd to the 4th quintile (Table 1).

**Table 1:** Baseline characteristics of participants overall and by quintiles of the FSAm-NPS DI, EPIC cohort, 1992–2014.

	All	Sex-specific quintiles of the FSAm-NPS DI score				
	(*n* = 471,495)	Q1 (*n* = 94,323)	Q2 (*n* = 94,341)	Q3 (*n* = 94,375)	Q4 (*n* = 94,278)	Q5 (*n* = 94,178)
	N (%)^a^ Mean ± SD	N (%)^a^ Mean ± SD	N (%) Mean ± SD	N (%) Mean ± SD	N (%) Mean ± SD	N (%) Mean ± SD
FSAm-NPS DI	6.0 ± 2.1	3.0 ± 1.0	4.8 ± 0.36	5.9 ± 0.33	7.1 ± 0.37	8.9 ± 1.1
Age, years	51.2 ± 9.9	51.6 ± 10.1	51.1 ± 9.8	50.8 ± 9.8	51.1 ± 9.9	51.2 ± 10.1
Sex						
Male	140,729 (29.8)	28,165 (29.9)	28,164 (29.8)	28,170 (29.8)	28,141 (29.8)	28,089 (29.8)
Female	330,766 (70.1)	66,158 (70.1)	66,177 (70.1)	66,205 (70.1)	66,137 (70.1)	66,089 (70.2)
Country						
Denmark	54,241 (11.5)	8,197 (8.7)	10,164 (10.8)	12,043 (12.8)	12,841 (13.6)	10,996 (11.7)
France	66,766 (14.2)	2,295 (2.4)	6,173 (6.5)	11,956 (12.7)	19,976 (21.2)	26,366 (28.0)
Greece	25,868 (5.5)	10,486 (11.1)	9,775 (10.4)	4,196 (4.4)	1,171 (1.2)	240 (0.25)
Germany	48,066 (10.2)	4,254 (4.5)	7,054 (7.5)	10,281 (10.9)	12,841 (13.6)	13,636 (14.5)
Italy	44,125 (9.4)	9,367 (9.9)	13,487 (14.3)	10,819 (11.5)	7,005 (7.4)	3,447 (3.7)
Norway	33,691 (7.1)	10,273 (10.9)	10,189 (10.8)	7,490 (7.9)	4,079 (4.3)	1,660 (1.8)
Spain	39,744 (8.4)	21,356 (22.6)	8,615 (9.1)	5,011 (5.3)	2,905 (3.1)	1,857 (2.0)
Sweden	48,078 (10.2)	7,176 (7.6)	8,966 (9.5)	9,954 (10.5)	10,277 (10.9)	11,705 (12.4)
The Netherlands	36,211 (7.7)	3,469 (3.7)	7,268 (7.7)	9,640 (10.2)	9,524 (10.1)	6,310 (6.7)
United Kingdom	74,705 (15.8)	17,450 (18.5)	12,650 (13.4)	12,985 (13.8)	13,659 (14.5)	17,961 (19.1)
Educational level^b^						
Longer education (including university degree), yes	112,434 (23.8)	19,000 (20.1)	20,592 (21.8)	23,109 (24.5)	25,162 (26.7)	24,571 (26.1)
Smoking status						
Never	203,399 (43.1)	46,448 (49.2)	41,869 (44.4)	39,852 (42.2)	38,556 (40.9)	36,674 (38.9)
Current	131,993 (28.0)	20,653 (21.9)	24,789 (26.3)	26,703 (28.3)	28,443 (30.2)	31,405 (33.3)
Former	120,577 (25.6)	24,486 (26.0)	24,619 (26.1)	24,648 (26.1)	24,102 (25.6)	22,722 (24.1)
Current/Former, missing	7,682 (1.6)	1,145 (1.2)	1,352 (1.4)	1,611 (1.7)	1,785 (1.9)	1,789 (1.9)
Unknown	7,844 (1.7)	1,591 (1.7)	1,712 (1.8)	1,561 (1.6)	1,392 (1.5)	1,588 (1.7)
Physical activity (Cambridge index)						
Inactive	98,710 (20.9)	24,186 (25.6)	20,624 (21.9)	18,049 (19.1)	17,103 (18.1)	18,748 (19.9)
Moderately inactive	155,211 (32.9)	28,293 (30.0)	29,821 (31.6)	31,184 (33.0)	32,719 (34.7)	33,194 (35.2)
Moderately active	124,377 (26.4)	23,994 (25.4)	25,415 (26.9)	25,172 (26.7)	25,086 (26.6)	24,710 (26.2)
Active	84,444 (17.9)	16,531 (17.5)	16,721 (17.7)	17,981 (19.0)	17,353 (18.4)	15,858 (16.8)
Missing	8,753 (1.9)	1,319 (1.4)	1,760 (1.9)	1,989 (2.1)	2,017 (2.1)	1,668 (1.8)
BMI^c^, kg/m^2^	25.4 ± 4.3	26.3 ± 4.5	25.8 ± 4.3	25.4 ± 4.2	25.0 ± 4.1	24.7 ± 4.1
Height^c^, cm	166.0 ± 9.0	164.5 ± 8.8	165.6 ± 9.0	166.4 ± 9.0	166.7 ± 9.0	166.7 ± 8.9
Family history of breast cancer, yes^d^	14,171 (3.0)	2,600 (18.3)	2,608 (18.4)	2,857 (20.2)	3,030 (21.4)	3,076 (21.7)
Family history of colorectal cancer, yes^d^	9,641 (2.0)	1,906 (19.8)	1,355 (14.0)	1,609 (16.7)	2,106 (21.8)	2,665 (27.6)
Energy intake, kcal/d^e^	1,997 (1,631–2,437)	1,750 (1,435–2,152)	1,905 (1,573–2,320)	1,988 (1,647–2,399)	2,094 (1,738–2,508)	2,256 (1,865–2,708)
Alcohol intake, g/d^e^	5.3 (0.93–14.9)	2.9 (0.35–11.9)	4.6 (0.82–13.3)	5.6 (1.1–15.2)	6.7 (1.5–16.5)	6.9 (1.5–17.1)
Dietary fibres intake, g/d^e^	21.8 (17.4–27.0)	24.2 (19.4–30.4)	22.5 (18.1–27.6)	21.7 (17.5–26.7)	21.2 (16.9–26.0)	19.9 (15.7–24.5)
Vegetables intake, g/d^e^	175.4 (109.9–276.6)	219.6 (134.3–340.8)	184.0 (115.7–294.1)	166.3 (107.1–260.3)	160.1 (103.7–248.3)	156.3 (98.0–242.1)
Fruits, nuts and seeds intake, g/d^e^	200.6 (111.6–322.3)	288.1 (174.3–436.0)	235.6 (132.6–356.9)	195.1 (111.6–308.9)	171.5 (98.5–272.3)	143.2 (79.8–233.1)
Dairy products intake, g/d^e^	277.2 (160.7–444.7)	267.5 (144.7–445.2)	282.4 (163.1–461.6)	293.6 (173.0–464.7)	284.4 (168.3–445.8)	258.7 (153.1–401.2)
Fish and shellfish intake, g/d^e^	28.0 (13.8–49.7)	32.9 (15.0–63.5)	28.5 (14.4–52.9)	27.3 (13.6–48.6)	26.4 (13.0–44.7)	25.3 (12.6–42.2)
Red meat intake, g/d^e^	34.8 (16.1–63.1)	26.5 (10.1–50.4)	34.4 (16.7–60.8)	37.5 (18.0–66.3)	40.3 (19.0–69.3)	37.0 (17.4–66.4)
Poultry intake, g/d^e^	15.0 (6.0–27.3)	16.1 (6.3–35.4)	15.9 (6.4–28.1)	15.0 (6.3–25.9)	13.7 (5.2–24.6)	11.8 (3.2–22.5)
Processed meat intake, g/d^e^	24.2 (10.5–43.8)	12.9 (3.1–27.4)	19.8 (7.6–36.3)	25.5 (12.4–43.8)	30.5 (16.1–50.9)	35.8 (18.6–60.1)
Age at menarche (years)^f^						
≤12	116,661 (35.3)	23,724 (35.9)	23,455 (35.4)	23,186 (35.0)	23,070 (34.9)	23,226 (35.1)
13–14	152,508 (46.1)	29,612 (44.8)	30,254 (45.7)	30,739 (46.4)	30,922 (46.7)	30,981 (46.9)
≥15	50,873 (15.4)	10,306 (15.6)	10,018 (15.1)	10,023 (15.1)	10,265 (15.5)	10,261 (15.5)
Missing	10,724 (3.2)	2,516 (3.8)	2,450 (3.7)	2,257 (3.4)	1,880 (2.8)	1,621 (2.4)
Age at first full-term pregnancy (years)^f^						
Nulliparous	47,901 (14.5)	10,683 (16.1)	9,057 (13.7)	9,261 (14.0)	9,108 (13.8)	9,792 (14.8)
≤21	60,915 (18.4)	12,644 (19.1)	12,765 (19.3)	12,246 (18.5)	11,623 (17.6)	11,637 (17.6)
22–30	180,029 (54.4)	34,969 (52.9)	36,022 (54.4)	36,160 (54.6)	36,786 (55.6)	36,092 (54.6)
>30	27,077 (8.2)	5,120 (7.7)	5,485 (8.3)	5,573 (8.4)	5,627 (8.5)	5,272 (8.0)
Missing	14,844 (4.5)	2,742 (4.1)	2,848 (4.3)	2,965 (4.5)	2,993 (4.5)	3,296 (5.0)
Menopausal status^f^						
Premenopause	115,631 (35.0)	22,199 (33.5)	23,011 (34.8)	23,686 (35.8)	23,177 (35.0)	23,558 (35.6)
Perimenopause	63,242 (19.1)	11,256 (17.0)	12,297 (18.6)	12,650 (19.1)	13,272 (20.1)	13,767 (20.8)
Postmenopause	142,368 (43.0)	30,326 (45.8)	28,833 (43.6)	28,011 (42.3)	27,959 (42.3)	27,239 (41.2)
Surgical postmenopause	9,525 (2.9)	2,377 (3.6)	2,036 (3.1)	1,858 (2.8)	1,729 (2.6)	1,525 (2.3)
Ever use of oral contraception (yes)^f^	189,288 (57.2)	32,555 (49.2)	34,986 (52.9)	38,689 (58.4)	41,060 (62.1)	41,998 (63.5)
Ever use of hormonal treatment for menopause (yes)^f^	79,929 (24.2)	14,562 (22.0)	15,275 (23.1)	16,478 (24.9)	17,056 (25.8)	16,558 (25.0)

^a^ Percentages are given in column.

^b^ Not specified for *N* = 16,701 (3.5%).

^c^ Missing BMI for *N* = 83,938 (17.8%), missing height for 82,875 (17.6%). When missing, height and weight were imputed with centre-, age-, and gender-specific average values.

^d^ Among first-degree relatives.

^e^ Values are median (interquartile range) for all dietary variables.

^f^ In women only.

**Abbreviations:** BMI, body mass index; DI, Dietary Index; EPIC, European Prospective Investigation into Cancer and Nutrition; FSAm-NPS, Nutrient Profiling System of the British Food Standards Agency (modified version).

Associations between the FSAm-NPS DI (continuous score and sex-specific quintiles) and cancer risk for different cancer types are displayed in Table 2 (overall) and Table 3 (by sex).

**Table 2:** Associations between the FSAm-NPS DI and cancer risk (total cancer and specific cancer types), from multivariable Cox proportional hazards models, EPIC cohort, 1992–2014.

	FSAm-NPS DI							
	Per 2-point increment	*P*-trend	Q1	Q2	Q3	Q4	Q5	*P*-trend
**FSAm-NPS DI range (men/women)**			−6.0–4.3/ −4.3–4.1	4.3–5.5/ 4.1–5.3	5.5–6.6/ 5.3–6.4	6.6–7.9/ 6.4–7.7	7.9–17.6/ 7.7–18.9	
**Total cancer**								
All (cases/person-years)	*49*,*794/6*,*635*,*062*		*9454/1*,*360*,*371*	*9482/1*,*327*,*943*	*9865/1*,*326*,*951*	*10*,*371/1*,*315*,*230*	*10*,*622/1*,*304*,*567*	
Sex-adjusted model—HR (95% CI)^a^	1.04 (1.03–1.05)	<0.001	1.00 (ref)	1.03 (1.00–1.06)	1.05 (1.02–1.08)	1.09 (1.05–1.12)	1.12 (1.08–1.15)	<0.001
Multi-adjusted model 1—HR (95% CI)^b^	1.02 (1.01–1.03)	<0.001	1.00 (ref)	1.02 (0.99–1.05)	1.03 (1.00–1.06)	1.06 (1.03–1.09)	1.07 (1.03–1.10)	<0.001
**Colorectal cancer**								
All (cases/person-years)	*5806/6*,*639*,*343*		*1144/1*,*361*,*188*	*1150/1*,*328*,*771*	*1152/1*,*327*,*731*	*1195/1*,*316*,*126*	*1165/1*,*305*,*527*	
Sex-adjusted model—HR (95% CI)	1.03 (1.00–1.06)	0.03	1.00 (ref)	1.07 (0.99–1.17)	1.07 (0.98–1.17)	1.11 (1.02–1.22)	1.11 (1.01–1.21)	0.02
Multi-adjusted model 1—HR (95% CI)	1.03 (1.00–1.06)	0.03	1.00 (ref)	1.07 (0.99–1.17)	1.07 (0.98–1.17)	1.12 (1.02–1.22)	1.11 (1.01–1.22)	0.02
**Bladder cancer**								
All (cases/person-years)	*1382/6*,*639*,*748*		*278/1*,*361*,*289*	*243/1*,*328*,*835*	*289/1*,*327*,*804*	*270/1*,*316*,*200*	*302/1*,*305*,*620*	
Sex-adjusted model—HR (95% CI)	1.06 (1.00–1.12)	0.04	1.00 (ref)	0.97 (0.81–1.16)	1.18 (0.98–1.40)	1.09 (0.90–1.31)	1.15 (0.96–1.39)	0.08
Multi-adjusted model 1—HR (95% CI)	1.02 (0.96–1.08)	0.6	1.00 (ref)	0.96 (0.80–1.14)	1.14 (0.95–1.36)	1.03 (0.85–1.24)	1.03 (0.85–1.25)	0.6
**Kidney cancer**								
All (cases/person-years)	*926/6*,*639*,*740*		*211/1*,*361*,*288*	*155/1*,*328*,*832*	*178/1*,*327*,*788*	*181/1*,*316*,*203*	*201/1*,*305*,*629*	
Sex-adjusted model—HR (95% CI)	1.09 (1.02–1.16)	0.02	1.00 (ref)	0.78 (0.63–0.97)	0.94 (0.76–1.16)	1.02 (0.82–1.26)	1.22 (0.98–1.52)	0.01
Multi-adjusted model 1—HR (95% CI)	1.07 (1.00–1.15)	0.06	1.00 (ref)	0.78 (0.63–0.96)	0.93 (0.75–1.15)	0.99 (0.80–1.24)	1.17 (0.93–1.46)	0.04
**Upper aerodigestive tract cancers**^**c**^								
All (cases/person-years)	*1*,*176/6*,*639*,*705*		*219/1*,*361*,*297*	*198/1*,*328*,*828*	*228/1*,*327*,*796*	*227/1*,*316*,*185*	*304/1*,*305*,*600*	
Sex-adjusted model—HR (95% CI)	1.16 (1.09–1.23)	<0.001	1.00 (ref)	1.00 (0.82–1.22)	1.13 (0.93–1.38)	1.11 (0.91–1.36)	1.52 (1.25–1.84)	<0.001
Multi-adjusted model 1—HR (95% CI)	1.07 (1.01–1.14)	0.03	1.00 (ref)	0.96 (0.78–1.17)	1.04 (0.85–1.27)	0.98 (0.79–1.20)	1.21 (0.99–1.48)	0.06
**Lung cancer**								
All (cases/person-years)	*3654/6*,*639*,*528*		*640/1*,*361*,*259*	*684/1*,*328*,*795*	*702/1*,*327*,*764*	*782/1*,*316*,*159*	*846/1*,*305*,*551*	
Sex-adjusted model—HR (95% CI)	1.16 (1.12–1.20)	<0.001	1.00 (ref)	1.11 (0.99–1.24)	1.17 (1.04–1.31)	1.34 (1.19–1.50)	1.57 (1.40–1.76)	<0.001
Multi-adjusted model 1—HR (95% CI)	1.01 (0.97–1.04)	0.7	1.00 (ref)	1.05 (0.94–1.17)	1.03 (0.92–1.16)	1.09 (0.97–1.22)	1.06 (0.94–1.20)	0.3
**Stomach cancer**								
All (cases/person-years)	*963/6*,*639*,*770*		*216/1*,*361*,*290*	*200/1*,*328*,*838*	*185/1*,*327*,*802*	*165/1*,*316*,*207*	*197/1*,*305*,*631*	
Sex-adjusted model—HR (95% CI)	1.13 (1.06–1.21)	0.0004	1.00 (ref)	1.07 (0.88–1.31)	1.10 (0.89–1.36)	1.08 (0.86–1.34)	1.39 (1.12–1.74)	0.01
Multi-adjusted model 1—HR (95% CI)	1.10 (1.02–1.18)	0.01	1.00 (ref)	1.06 (0.87–1.29)	1.08 (0.87–1.33)	1.02 (0.82–1.29)	1.25 (0.99–1.58)	0.1
**Pancreas cancer**								
All (cases/person-years)	*1244/6*,*639*,*760*		*260/1*,*361*,*295*	*240/1*,*328*,*830*	*251/1*,*327*,*800*	*254/1*,*316*,*205*	*239/1*,*305*,*630*	
Sex-adjusted model—HR (95% CI)	1.00 (0.95–1.06)	0.9	1.00 (ref)	0.96 (0.80–1.15)	1.01 (0.84–1.22)	1.04 (0.86–1.25)	1.02 (0.84–1.24)	0.6
Multi-adjusted model 1—HR (95% CI)	0.98 (0.92–1.04)	0.4	1.00 (ref)	0.94 (0.79–1.13)	0.98 (0.82–1.18)	0.99 (0.82–1.20)	0.94 (0.77–1.15)	0.7
**Liver cancer**								
All (cases/person-years)	*338/6*,*639*,*776*		*71/1*,*361*,*289*	*64/1*,*328*,*835*	*60/1*,*327*,*811*	*70/1*,*316*,*211*	*73/1*,*305*,*629*	
Sex-adjusted model 1—HR (95% CI)	1.10 (0.98–1.24)	0.1	1.00 (ref)	0.96 (0.68–1.36)	1.02 (0.71–1.47)	1.26 (0.87–1.81)	1.36 (0.94–1.98)	0.046
Multi-adjusted model 1—HR (95% CI)	1.05 (0.93–1.18)	0.5	1.00 (ref)	0.92 (0.65–1.31)	0.95 (0.66–1.38)	1.15 (0.79–1.67)	1.18 (0.80–1.74)	0.2
**Prostate cancer**								
Men (cases/person-years)	*6745/1*,*978*,*301*		*1192/400*,*545*	*1162/393*,*399*	*1365/397*,*646*	*1471/395*,*278*	*1555/391*,*434*	
Unadjusted model—HR (95% CI)^d^	1.02 (1.00–1.05)	0.1	1.00 (ref)	0.99 (0.91–1.07)	1.05 (0.96–1.14)	1.05 (0.97–1.15)	1.06 (0.97–1.15)	0.08
Multi-adjusted model 1—HR (95% CI)	1.03 (1.00–1.06)	0.04	1.00 (ref)	0.99 (0.91–1.07)	1.05 (0.97–1.15)	1.06 (0.97–1.16)	1.07 (0.98–1.17)	0.04
**Breast cancer**								
Women (cases/person-years)	*12*,*063/4*,*659*,*777*		*2093/960*,*453*	*2303/935*,*107*	*2403/929*,*855*	*2628/920*,*557*	*2636/913*,*805*	
Unadjusted model—HR (95% CI)	1.03 (1.01–1.05)	0.01	1.00 (ref)	1.05 (0.99–1.12)	1.04 (0.98–1.11)	1.09 (1.02–1.16)	1.08 (1.01–1.15)	0.01
Multi-adjusted model 2—HR (95% CI)^e^	1.02 (1.00–1.04)	0.05	1.00 (ref)	1.04 (0.98–1.11)	1.03 (0.97–1.10)	1.07 (1.01–1.14)	1.06 (0.99–1.14)	0.05
**Endometrial cancer**^**f**^								
Women (cases/person-years)	*1*,*763/4*,*529*,*816*		*401/926*,*746*	*377/907*,*086*	*344/904*,*957*	*361/897*,*398*	*280/893*,*630*	
Unadjusted model—HR (95% CI)	0.95 (0.91–1.00)	0.06	1.00 (ref)	1.00 (0.87–1.16)	0.94 (0.81–1.09)	1.03 (0.88–1.20)	0.85 (0.72–1.00)	0.1
Multi-adjusted model 2—HR (95% CI)	0.98 (0.93–1.03)	0.4	1.00 (ref)	1.02 (0.88–1.18)	0.98 (0.84–1.14)	1.09 (0.93–1.27)	0.91 (0.76–1.08)	0.6
**Cervical cancer**^**f**^								
Women (cases/person-years)	*305/4*,*529*,*956*		*66/926*,*769*	*71/907*,*109*	*62/904*,*989*	*60/897*,*436*	*46/893*,*652*	
Unadjusted model—HR (95% CI)	1.04 (0.92–1.17)	0.5	1.00 (ref)	1.18 (0.84–1.66)	1.11 (0.77–1.59)	1.20 (0.83–1.75)	1.01 (0.67–1.52)	0.8
Multi-adjusted model 2—HR (95% CI)	1.05 (0.93–1.18)	0.5	1.00 (ref)	1.20 (0.85–1.70)	1.12 (0.78–1.62)	1.23 (0.84–1.80)	1.02 (0.67–1.56)	0.8
**Ovary cancer**^**f**^								
Women (cases/person-years)	*1*,*273/4*,*529*,*820*		*268/926*,*740*	*235/907*,*088*	*264/904*,*948*	*253/897*,*412*	*253/893*,*632*	
Unadjusted model—HR (95% CI)	1.04 (0.98–1.10)	0.2	1.00 (ref)	0.92 (0.77–1.11)	1.06 (0.89–1.27)	1.05 (0.87–1.26)	1.08 (0.89–1.30)	0.2
Multi-adjusted model 2—HR (95% CI)	1.04 (0.98–1.11)	0.2	1.00 (ref)	0.93 (0.78–1.11)	1.07 (0.90–1.28)	1.06 (0.88–1.28)	1.08 (0.89–1.31)	0.2

^a^ Sex-adjusted models were stratified for centre and age at recruitment (1-y intervals, time-scale) and adjusted for sex.

^b^ Multi-adjusted model 1 was stratified for centre and age at recruitment (1-y intervals, time-scale) and adjusted for sex, BMI (continuous), height (continuous),baseline alcohol intake (g/d), physical activity (Cambridge index: active; moderately active; moderately inactive; inactive; missing), smoking status and intensity of smoking (current, 1–15 cigarettes/d; current, 16–25 cigarettes/d; current, 26+ cigarettes/d; current, pipe/cigar/occasional; current/former, missing; former, quit 11–20 y; former, quit 20+ y; former, quit ≤10 y; never; unknown), family history of breast cancer (total and breast cancer models), family history of colorectal cancer (total and colorectal cancer models), educational level (longer education [including university degree]; secondary school; primary school completed; not specified), baseline energy intake (kcal/d).

^c^ Upper aerodigestive tract cancers: cancers of the oral cavity, oropharynx, hypopharynx, larynx, and oesophagus.

^d^ Unadjusted models were stratified for centre and age at recruitment (1-y intervals, time-scale).

^e^ Multi-adjusted model 2: Multi-adjusted model 1 further adjusted for an interaction term between BMI and menopausal status, menopausal status (premenopausal, perimenopausal, postmenopausal, surgical), ever use of oral contraception (yes, no, missing), ever use of hormonal treatment for menopause (yes, no, missing), age at menarche (≤12 y, 13–14 y, ≥15 y, missing), age at first full-term pregnancy (nulliparous, ≤21 y, 22–30 y, >30 y, missing) and age at menopause (<50 y, ≥50 y).

^f^ Women who declared a surgical menopause at baseline were excluded.

**Abbreviations:** BMI, body mass index; DI, Dietary Index; EPIC, European Prospective Investigation into Cancer and Nutrition; FSAm-NPS, Nutrient Profiling System of the British Food Standards Agency (modified version); HR, hazard ratio.

**Table 3:** Associations between the FSAm-NPS DI and cancer risk (total cancer and specific cancer types) by sex strata, from multivariable Cox proportional hazards models, EPIC cohort, 1992–2014.

	FSAm-NPS DI							
	2-point increment	*P*-trend	Q1	Q2	Q3	Q4	Q5	*P*-trend
**FSAm-NPS DI range (men/women)**			−6.0–4.3/ −4.3–4.1	4.3–5.5/ 4.1–5.3	5.5–6.6/ 5.3–6.4	6.6–7.9/ 6.4–7.7	7.9–17.6/ 7.7–18.9	
**Total cancer**								
*P*-interaction		0.4						0.6
Men (cases/person-years)	*19*,*711/1*,*977*,*015*		*3585/400*,*287*	*3494/393*,*163*	*3838/397*,*419*	*4259/395*,*015*	*4535/391*,*131*	
Unadjusted model—HR (95% CI)^a^	1.05 (1.03–1.06)	<0.001	1.00 (ref)	1.03 (0.98–1.08)	1.07 (1.02–1.12)	1.12 (1.07–1.18)	1.15 (1.09–1.20)	<0.001
Multi-adjusted model—HR (95% CI)^b^	1.03 (1.01–1.04)	0.002	1.00 (ref)	1.01 (0.97–1.06)	1.04 (0.99–1.10)	1.08 (1.03–1.14)	1.07 (1.02–1.13)	0.001
Women (cases/person-years)	*30*,*083/4*,*658*,*047*		*5869/960*,*083*	*5988/934*,*780*	*6027/929*,*532*	*6112/920*,*215*	*6087/913*,*437*	
Unadjusted model—HR (95% CI)	1.03 (1.02–1.05)	<0.001	1.00 (ref)	1.03 (1.00–1.07)	1.04 (1.00–1.08)	1.07 (1.03–1.11)	1.11 (1.06–1.15)	<0.001
Multi-adjusted model—HR (95% CI)	1.02 (1.01–1.03)	0.001	1.00 (ref)	1.02 (0.99–1.06)	1.03 (0.99–1.06)	1.05 (1.01–1.09)	1.07 (1.03–1.11)	0.001
**Colorectal cancer**								
*P*-interaction		0.04						0.04
Men (cases/person-years)	*2506/1*,*978*,*384*		*489/400*,*529*	*463/393*,*421*	*485/397*,*669*	*542/395*,*305*	*527/391*,*460*	
Unadjusted model—HR (95% CI)^a^	1.02 (0.98–1.07)	0.3	1.00 (ref)	1.05 (0.92–1.20)	1.05 (0.92–1.21)	1.12 (0.98–1.29)	1.06 (0.92–1.22)	0.3
Multi-adjusted model—HR (95% CI)^b^	1.03 (0.98–1.07)	0.2	1.00 (ref)	1.05 (0.92–1.20)	1.05 (0.91–1.20)	1.12 (0.98–1.29)	1.07 (0.92–1.24)	0.2
Women (cases/person-years)	*3300/4*,*660*,*959*		*655/960*,*658*	*687/935*,*350*	*667/930*,*062*	*653/920*,*822*	*638/914*,*067*	
Unadjusted model—HR (95% CI)	1.05 (1.01–1.08)	0.01	1.00 (ref)	1.10 (0.98–1.22)	1.10 (0.98–1.23)	1.12 (1.00–1.26)	1.18 (1.05–1.33)	0.01
Multi-adjusted model—HR (95% CI)	1.04 (1.00–1.08)	0.03	1.00 (ref)	1.09 (0.98–1.22)	1.10 (0.98–1.23)	1.12 (0.99–1.26)	1.17 (1.03–1.32)	0.02
**Bladder cancer**								
*P*-interaction		0.8						0.6
Men (cases/person-years)	*987/1*,*978*,*521*		*197/400*,*572*	*170/393*,*445*	*199/397*,*688*	*202/395*,*333*	*219/391*,*484*	
Unadjusted model—HR (95% CI)^a^	1.08 (1.01–1.16)	0.02	1.00 (ref)	1.00 (0.81–1.24)	1.20 (0.96–1.49)	1.20 (0.96–1.50)	1.20 (0.96–1.50)	0.05
Multi-adjusted model—HR (95% CI)^b^	1.04 (0.97–1.11)	0.3	1.00 (ref)	0.98 (0.79–1.22)	1.14 (0.92–1.42)	1.12 (0.89–1.40)	1.05 (0.83–1.33)	0.5
Women (cases/person-years)	*395/4*,*661*,*227*		*81/960*,*717*	*73/935*,*390*	*90/930*,*116*	*68/920*,*868*	*83/914*,*136*	
Unadjusted model—HR (95% CI)	1.04 (0.94–1.16)	0.4	1.00 (ref)	0.96 (0.70–1.33)	1.24 (0.91–1.70)	0.96 (0.68–1.36)	1.19 (0.85–1.67)	0.4
Multi-adjusted model—HR (95% CI)	1.01 (0.91–1.12)	0.9	1.00 (ref)	0.94 (0.68–1.30)	1.20 (0.87–1.65)	0.91 (0.64–1.28)	1.07 (0.75–1.52)	0.8
**Kidney cancer**								
*P*-interaction		0.4						0.4
Men (cases/person-years)	*522/1*,*978*,*557*		*119/400*,*579*	*76/393*,*450*	*90/397*,*692*	*112/395*,*339*	*125/391*,*497*	
Unadjusted model—HR (95% CI)^a^	1.08 (0.99–1.18)	0.1	1.00 (ref)	0.70 (0.52–0.95)	0.84 (0.62–1.13)	1.02 (0.76–1.38)	1.13 (0.84–1.52)	0.07
Multi-adjusted model—HR (95% CI)^b^	1.05 (0.96–1.16)	0.3	1.00 (ref)	0.68 (0.51–0.92)	0.80 (0.59–1.08)	0.97 (0.72–1.31)	1.04 (0.77–1.42)	0.2
Women (cases/person-years)	*404/4*,*661*,*183*		*92/960*,*709*	*79/935*,*383*	*88/930*,*096*	*69/920*,*864*	*76/914*,*131*	
Unadjusted model—HR (95% CI)	1.11 (1.00–1.24)	0.04	1.00 (ref)	0.90 (0.66–1.23)	1.12 (0.83–1.52)	1.03 (0.74–1.43)	1.41 (1.02–1.96)	0.04
Multi-adjusted model—HR (95% CI)	1.12 (1.01–1.25)	0.03	1.00 (ref)	0.92 (0.67–1.25)	1.15 (0.85–1.57)	1.06 (0.76–1.49)	1.45 (1.03–2.04)	0.03
**Upper aerodigestive tract cancers**^**c**^								
*P*-interaction		0.9						0.8
Men (cases/person-years)	*786/1*,*978*,*504*		*138/400*,*581*	*122/393*,*446*	*158/397*,*686*	*158/395*,*320*	*210/391*,*471*	
Unadjusted model—HR (95% CI)^a^	1.19 (1.11–1.28)	<0.001	1.00 (ref)	1.03 (0.80–1.33)	1.28 (1.00–1.64)	1.22 (0.94–1.58)	1.61 (1.26–2.07)	<0.001
Multi-adjusted model—HR (95% CI)^b^	1.09 (1.01–1.18)	0.02	1.00 (ref)	0.97 (0.75–1.26)	1.14 (0.89–1.47)	1.03 (0.79–1.34)	1.25 (0.96–1.62)	0.08
Women (cases/person-years)	*390/4*,*661*,*201*		*81/960*,*716*	*76/935*,*382*	*70/930*,*109*	*69/920*,*865*	*94/914*,*130*	
Unadjusted model—HR (95% CI)	1.11 (1.01–1.22)	0.04	1.00 (ref)	1.01 (0.73–1.38)	0.94 (0.67–1.30)	1.00 (0.71–1.40)	1.46 (1.06–2.00)	0.04
Multi-adjusted model—HR (95% CI)	1.03 (0.93–1.14)	0.6	1.00 (ref)	0.96 (0.70–1.32)	0.87 (0.62–1.21)	0.88 (0.63–1.24)	1.16 (0.83–1.62)	0.5
**Lung cancer**								
*P*-interaction		0.5						0.3
Men (cases/person-years)	*1876/1*,*978*,*425*		*297/400*,*564*	*336/393*,*426*	*343/397*,*670*	*415/395*,*313*	*485/391*,*452*	
Unadjusted model—HR (95% CI)^a^	1.21 (1.15–1.27)	<0.001	1.00 (ref)	1.29 (1.10–1.52)	1.39 (1.17–1.65)	1.65 (1.39–1.96)	1.94 (1.64–2.31)	<0.001
Multi-adjusted model—HR (95% CI)^b^	1.04 (0.99–1.09)	0.2	1.00 (ref)	1.21 (1.02–1.43)	1.21 (1.02–1.44)	1.31 (1.10–1.60)	1.26 (1.06–1.51)	0.02
Women (cases/person-years)	*1778/4*,*661*,*103*		*343/960*,*695*	*348/935*,*369*	*359/930*,*094*	*367/920*,*846*	*361/914*,*099*	
Unadjusted model—HR (95% CI)	1.14 (1.09–1.20)	<0.001	1.00 (ref)	1.01 (0.87–1.18)	1.10 (0.94–1.28)	1.24 (1.06–1.44)	1.46 (1.24–1.71)	<0.001
Multi-adjusted model—HR (95% CI)	0.99 (0.95–1.04)	0.8	1.00 (ref)	0.94 (0.80–1.09)	0.95 (0.81–1.11)	0.99 (0.84–1.16)	0.97 (0.82–1.14)	0.9
**Stomach cancer**								
*P*-interaction		0.6						0.9
Men (cases/person-years)	*535/1*,*978*,*569*		*115/400*,*585*	*106/393*,*454*	*101/397*,*693*	*97/395*,*342*	*116/391*,*495*	
Unadjusted model—HR (95% CI)^a^	1.11 (1.01–1.22)	0.03	1.00 (ref)	1.14 (0.86–1.50)	1.20 (0.89–1.61)	1.18 (0.87–1.61)	1.39 (1.02–1.89)	0.06
Multi-adjusted model—HR (95% CI)^b^	1.08 (0.98–1.19)	0.1	1.00 (ref)	1.14 (0.86–1.50)	1.17 (0.87–1.58)	1.13 (0.82–1.55)	1.26 (0.91–1.73)	0.2
Women (cases/person-years)	*428/4*,*661*,*201*		*101/960*,*706*	*94/935*,*384*	*84/930*,*109*	*68/920*,*866*	*81/914*,*136*	
Unadjusted model—HR (95% CI)	1.17 (1.06–1.30)	0.002	1.00 (ref)	1.03 (0.77–1.37)	1.04 (0.77–1.41)	1.00 (0.72–1.39)	1.49 (1.08–2.06)	0.05
Multi-adjusted model—HR (95% CI)	1.12 (1.01–1.24)	0.04	1.00 (ref)	0.99 (0.74–1.33)	0.97 (0.72–1.33)	0.91 (0.65–1.27)	1.27 (0.91–1.79)	0.4
**Pancreas cancer**								
*P*-interaction		0.9						0.8
Men (cases/person-years)	*526/1*,*978*,*561*		*98/400*,*580*	*92/393*,*450*	*110/397*,*696*	*121/395*,*338*	*105/391*,*496*	
Unadjusted model—HR (95% CI)^a^	1.01 (0.92–1.11)	0.8	1.00 (ref)	0.92 (0.68–1.23)	1.05 (0.78–1.42)	1.10 (0.82–1.48)	0.97 (0.71–1.33)	0.7
Multi-adjusted model—HR (95% CI)^b^	0.98 (0.89–1.08)	0.7	1.00 (ref)	0.89 (0.66–1.20)	1.01 (0.75–1.37)	1.04 (0.77–1.41)	0.89 (0.64–1.22)	0.8
Women (cases/person-years)	*718/4*,*661*,*199*		*162/960*,*715*	*148/935*,*380*	*141/930*,*103*	*133/920*,*867*	*134/914*,*134*	
Unadjusted model—HR (95% CI)	1.01 (0.93–1.09)	0.9	1.00 (ref)	0.98 (0.78–1.23)	0.99 (0.78–1.25)	0.99 (0.78–1.27)	1.09 (0.85–1.40)	0.5
Multi-adjusted model—HR (95% CI)	0.97 (0.90–1.06)	0.5	1.00 (ref)	0.96 (0.77–1.21)	0.96 (0.76–1.22)	0.95 (0.74–1.22)	1.00 (0.77–1.30)	0.9
**Liver cancer**								
P-interaction		0.07						0.04
Men (cases/person-years)	*210/1*,*978*,*566*		*48/400*,*584*	*36/393*,*452*	*39/397*,*696*	*44/395*,*340*	*43/391*,*495*	
Unadjusted model—HR (95% CI)^a^	1.00 (0.86–1.16)	0.9	1.00 (ref)	0.79 (0.51–1.23)	0.91 (0.57–1.44)	0.97 (0.61–1.54)	0.93 (0.57–1.50)	0.9
Multi-adjusted model—HR (95% CI)^b^	0.94 (0.80–1.10)	0.4	1.00 (ref)	0.74 (0.47–1.17)	0.82 (0.51–1.32)	0.88 (0.54–1.42)	0.79 (0.48–1.30)	0.6
Women (cases/person-years)	*128/4*,*661*,*210*		*23/960*,*705*	*28/935*,*383*	*21/930*,*116*	*26/920*,*871*	*30/914*,*135*	
Unadjusted model—HR (95% CI)	1.30 (1.08–1.57)	0.007	1.00 (ref)	1.40 (0.79–2.48)	1.31 (0.70–2.45)	1.95 (1.06–3.60)	2.64 (1.42–4.89)	0.002
Multi-adjusted model—HR (95% CI)	1.24 (1.02–1.51)	0.03	1.00 (ref)	1.35 (0.76–2.41)	1.23 (0.65–2.31)	1.80 (0.96–3.34)	2.33 (1.23–4.43)	0.008

^a^ Unadjusted models were stratified for centre and age at recruitment (1-y intervals, time-scale).

^b^ Multi-adjusted models were stratified for centre and age at recruitment (1-y intervals, time-scale) and adjusted for BMI (continuous), height (continuous),baseline alcohol intake (g/d), physical activity (Cambridge index: active; moderately active; moderately inactive; inactive; missing), smoking status and intensity of smoking (current, 1–15 cigarettes/d; current, 16–25 cigarettes/d; current, 26+ cigarettes/d; current, pipe/cigar/occasional; current/former, missing; former, quit 11–20 y; former, quit 20+y; former, quit ≤10 y; never; unknown), family history of breast cancer (total and breast cancer models), family history of colorectal cancer (total and colorectal cancer models), educational level (longer education [including university degree]; secondary school; primary school completed; not specified), baseline energy intake (kcal/d) + (women) menopausal status (premenopausal, perimenopausal, postmenopausal, surgical), ever use of oral contraception (yes, no, missing), ever use of hormonal treatment for menopause (yes, no, missing).

^c^ Upper aerodigestive tract cancers: cancers of the oral cavity, oropharynx, hypopharynx, larynx, and oesophagus.

**Abbreviations:** BMI, body mass index; DI, Dietary Index; EPIC, European Prospective Investigation into Cancer and Nutrition; FSAm-NPS, Nutrient Profiling System of the British Food Standards Agency (modified version); HR, hazard ratio.

A higher FSAm-NPS DI score was associated with a higher risk of total cancer (HR_Q5_ versus _Q1_ = 1.07; 95% CI 1.03–1.10, *P*-trend < 0.001). The absolute rates in those with high and low FSAm-NPS DI scores were 81.4 (men: 115.9; women: 66.6) and 69.5 (men: 89.6; women: 61.1) cases per 10,000 person-years, respectively.

Regarding specific cancer types, a higher FSAm-NPS DI was associated with a higher risk of colorectal cancer (HR_Q5_ versus _Q1_ = 1.11 (1.01–1.22), *P*-trend = 0.02), especially in women (*P*-interaction = 0.04). A higher FSAm-NPS DI was also associated with a higher liver cancer risk in women (HR_Q5_ versus _Q1_ = 2.33 (1.23–4.43), *P*-trend = 0.008, *P*-interaction = 0.04) and a higher lung cancer risk in men (HR_Q5_ versus _Q1_ = 1.26 (1.06–1.51), *P*-trend = 0.02) although the interaction with sex was not significant for lung cancer (*P*-interaction = 0.3). If only borderline nonsignificant trends were observed when comparing the highest and the lowest quintiles of the FSAm-NPS DI, 2-point increment in the FSAm-NPS DI score was associated with higher risks of stomach cancer (HR per 2-point increment = 1.10 (1.02–1.18), *P*-trend = 0.01) and of cancers of the upper aerodigestive tract (HR per 2-point increment = 1.07 (1.01–1.14), *P*-trend = 0.03). A borderline significant association was also observed for kidney cancer (HR_Q5_ versus _Q1_ = 1.17 (0.93–1.46), *P*-trend = 0.04). No association was observed for cancers of the bladder (*P*-trend = 0.6) and pancreas (*P*-trend = 0.7).

For sex-specific cancers of the reproductive system, a higher FSAm-NPS DI score was associated with a higher risk of postmenopausal breast cancer (HR_Q5_ versus _Q1_ = 1.08 (1.00–1.16), *P*-trend = 0.03, S2 Table) and a borderline significant higher risk of prostate cancer (HR_Q5_ versus _Q1_ = 1.07 (0.98–1.17), *P*-trend = 0.04, Table 2). No association was detected for cancers of the endometrium, uterine cervix, or ovaries (Table 2).Similar results were observed for overall cancer risk when complete cases models were used (40,945 cases/5,201,091 person-years, HR_Q5_ versus _Q1_ = 1.07 (1.03–1.11), *P*-trend < 0.001) and when models were not adjusted for BMI (HR_Q5_ versus _Q1_ 95% CI 1.07 [1.03–1.10], *P*-trend < 0.001).

## Discussion

In this large multinational European cohort, participants with the highest FSAm-NPS DI scores, i.e., those consuming on average food products with a lower nutritional quality, were at higher risk of developing cancer overall. Stronger associations were observed for colorectal, upper aerodigestive tract, and stomach cancers, for lung cancer in men, and for liver and postmenopausal breast cancers in women.

To our knowledge, this study was the first effort to investigate the association between the FSAm-NPS DI and disease in a large European cohort. Consistent with our results, previous studies performed in the SU.VI.MAX and NutriNet-Santé cohorts reported higher risks for total and breast cancers with higher FSAm-NPS DI scores [29,30]. However, these studies exhibited limited statistical power to investigate the relationships for other specific cancer types.

With a different approach, using the original FSA-NPS score and the Ofcom regulation threshold [9] to categorize food/beverage as ‘healthier’ or ‘less healthy’, Masset and colleagues observed a lower all-cause and cancer mortality associated to the intake of a greater variety of ‘healthier’ food items in the Whitehall II cohort [42], and, recently, Mytton and colleagues observed a higher all-cause mortality associated to the consumption of ‘less healthy’ food items in EPIC-Norfolk [43].

The comparison between other dietary scores and the FSAm-NPS DI is not straightforward. Indeed, the FSAm-NPS DI is a dietary score based on a nutrient profiling system at the level of food products, obtained following a two-step process. First, all food and beverage items are assigned a score according to their nutritional quality (FSAm-NPS). Then, an individual index, the FSAm-NPS DI, is computed at the individual level (mainly for research purposes) by calculating a weighted mean of the FSAm-NPS scores of all food/beverages consumed by this individual. In contrast, usual dietary scores are obtained directly at the individual level, allocating points based on the consumption of foods/food groups or nutrients relevant for overall or specific chronic disease risk (e.g., Mediterranean diet score [44], WCRF/AICR adherence score [45], Alternate Healthy Eating Index [46]). Therefore, these scores relate more to individual eating behaviours than to the intrinsic nutritional quality of the foods consumed, with objective to add support to dietary recommendations and/or be a basis for dietary guidelines. The FSAm-NPS was not designed to find the best predictive score for cancer risk but rather to serve as a basis for food nutritional labelling (such as the Nutri-Score) and other public health nutritional policies (e.g., advertising regulation) in order to improve the prevention of a large range of chronic diseases. As such, it has to be easily computable by industrial and public stakeholders (thus including only items generally present in the nutritional facts of all food labels). Hence, our objective was not to compare the FSAm-NPS DI score to other existing dietary scores but to specifically assess the relevance of the use of the FSAm-NPS score to grade the nutritional quality of food products in the framework of public health policies aiming at reducing cancer risk.

To our knowledge, the Overall Nutritional Quality Index (ONQI-f) is the only other dietary score based on a nutrient profiling system at the food level that has been translated at the individual level and then studied in relation to health outcomes so far [47]. In a study performed in the Nurses’ Health Study and the Health Professionals Follow-up Study, Chiuve and colleagues observed that a higher ONQI-f (reflecting a higher overall nutritional quality of the diet), was associated with a lower risk of cardiovascular diseases, diabetes, and mortality but was not associated with cancer risk. Nonetheless, it is important to note that the ONQI-f is based on 30 nutrients (from macronutrients such as fat, protein, or glycaemic load to micronutrients such as folate, vitamin D, zinc, iron, or omega 3 fatty acids but also polyphenols [flavonoids]), among which few have shown a consistent association with cancer risk, which may have weakened its relevance for the cancer outcome.

In contrast, the FSAm-NPS score relies on a limited number of components for which information is readily available on food packaging; in addition, most of these components have been proposed to be involved in cancer development in epidemiological and mechanistic studies. Inverse associations have been observed between dietary fibre intake and colorectal [48,49] and breast [48] cancer risk, fruit and vegetable intakeand risk of cancers of the mouth/larynx/pharynx and lung [48,49], whereas positive associations have been observed between salt intake and stomach cancer risk [48,49] and sugar intake (as a contributor to glycaemic load) and endometrial cancer risk [49]. In addition, even though the evidence for an association between saturated fatty acid intake and breast cancer risk was classified as ‘limited-no conclusion’ in the last WCRF report, the corresponding meta-analysis did show a direct association [50]. Indirect associations may also be proposed between the FSAm-NPS components and cancer risk through an association with body fatness, a major risk factor for most cancer locations (oesophagus, stomach, pancreas, liver, colon-rectum, breast [postmenopausal], ovary, endometrium, prostate, and kidney) [48,49]. Indeed, FSAm-NPS components contribute to the energy density of foods, with energy, sugars, and saturated fatty acids as components of energy-dense foods and fibres and fruits and vegetables as components of low-energy foods.

In our study, nonsignificant results were observed for specific cancer types in men, women, or both, and most associations were weak compared to other studies exploring FSAm-NPS DI in relation with cancers (SU.VI.MAX: total cancer, 453 cases, HR_Q5_ versus _Q1_ = 1.34 [1.00–1.81]); NutriNet-Santé, breast cancer, 555 cases: HR_Q5_ versus _Q1_ = 1.52 [1.11–2.08] [29,30]). Although HR cannot be compared directly between our study and previous ones because of differences in population and methods, several hypotheses may be proposed to explain the rather weak associations observed here. Most centres participating in the EPIC cohort used an FFQ to assess dietary intakes. FFQs allow good estimations of usual dietary intakes but limit the discrimination of the nutritional quality of individual food products, especially when they are collapsed into aggregated food groups. The use of FFQs may have contributed to a less accurate estimation of the individual FSAm-NPS DI scores and thus to a dilution of the potential effect [51], with weaker associations than the ones that could have been observed with the use of other dietary assessment methods (e.g., repeated 24-h dietary records as used in the SU.VI.MAX and NutriNet-Santé cohorts). Differences in effect size between studies and between cancer locations could also be partially explained by the differences in the number of cases; fewer cases may lead to less-accurate estimates of HRs. Finally, the differences in effect size between cancer locations and the nonsignificant results observed for some cancer sites may illustrate true different susceptibilities of cancer types to nutritional factors. For example, WCRF and AICR estimated that 47% of colorectal cancer may be prevented with nutrition compared to 19% for pancreatic cancers [1].

Strengths of this study include its large sample size, its prospective design, its long follow-up, and the inclusion of participants from different European countries with standardised data collection, especially for diet, offering a broad perspective on nutritional quality of dietary intakes in Europe. However, some limitations should be acknowledged. First, caution is needed regarding the extrapolation of these results to the entire European population or to other populations or ethnicities worldwide since this study included volunteers from 10 European countries involved in a long-term cohort study investigating the association between nutrition and health, with overall more health-conscious behaviours compared to the general population. Therefore, unhealthy dietary behaviours may have been underrepresented in this study, which may have weakened the observed associations by inducing a smaller contrast between high and low scores. Furthermore, in our models, we included all the participants with available dietary intake data but with potential missing data on other covariates replaced with a ‘missing’ class or imputation. Although this may have induced some bias, a complete cases model would lead to a selection towards more compliant participants in an already health-conscious population. Still, sensitivity analyses with a complete cases model provided similar results. In addition, this study used a single assessment of dietary intakes at baseline. Although diet may change over time, it is usually hypothesized that this estimation reflects general eating behavior throughout middle-aged adult life [52]. Diet measurement instruments are built to capture the usual dietary intakes of an individual but are still subject to imprecision and inaccuracy. Finally, this study was based on an observational cohort. Thus, even though our models included a large range of confounding factors, residual confounding cannot be entirely ruled out.

In conclusion, the results of this observational study performed on a large European cohort with diverse profiles and nutritional habits, suggest that the consumption of food products with higher FSAm-NPS scores (reflecting a lower nutritional quality) is associated with a higher risk of developing cancer. These studies complement published or ongoing studies specifically assessing the perception and understanding of the Nutri-Score (derived from FSAm-NPS score) and its actual impact on food choices [13–22]. Overall, this adds support to the relevance of the FSAm-NPS as underlying nutrient profiling system for the simplified nutrition label Nutri-Score, but also for other public health nutritional measures aiming to influence the nutritional quality of food choices at a national and potentially supranational level. This should be considered for ongoing and future debates at the EU level regarding the implementation of a unique food labelling system on the front of pack of food products. To date, the FSAm-NPS is the most validated nutrient-profiling system and the easiest to compute, with a limited number of components that are readily available on food packaging and an open/published algorithm. Future comparative studies may be carried on if other nutrient profiling systems with similar characteristics, and a corresponding score derived at the individual level are to be proposed. Appending a nutritional label like the Nutri-Score would be an additional tool to the array of public health nutritional strategies. In particular, this would complement strategies setting the bases of a balanced diet mixing different types of food, by helping the consumers choose food products with a better nutritional profile, even among the same food category, and by highlighting food products for which a sensible consumption should be preferred.

## Supporting information

S1 Fig The Nutri-Score front-of-pack nutritional label (Santé Publique France). (PDF)

S2 Fig Participants’ flowchart, EPIC cohort, 1992–2014. EPIC, European Prospective Investigation into Cancer and Nutrition.
(PDF)

S1 Table Incident cancer cases and noncases by country, EPIC cohort, 1992–2014. EPIC, European Prospective Investigation into Cancer and Nutrition.
(PDF)

S2 Table Associations between the FSAm-NPS DI and risk for cancers of the female reproductive system, by menopausal status, from multivariable Cox proportional hazards models, EPIC cohort, 1992–2014. DI, Dietary Index; EPIC, European Prospective Investigation into Cancer and Nutrition; FSAm-NPS, Nutrient Profiling System of the British Food Standards Agency (modified version).
(PDF)

S1 Text FSAm-NPS score computation at food/beverage level, FSAm-NPS DI computation at individual level and link to the Nutri-Score (Santé Publique France). DI, Dietary Index; FSAm-NPS, Nutrient Profiling System of the British Food Standards Agency (modified version).
(PDF)

S2 Text Analysis plan extracted from the project protocol submitted for funding application. (PDF)

S1 STROBE Statement (PDF)

## Abbreviations

AICR: American Institute for Cancer Research
BMI: body mass index
DI: Dietary Index
EPIC: European Prospective Investigation into Cancer and Nutrition
EU: European Union
FFQ: food frequency questionnaire
FSA-NPS: Nutrient Profiling System of the British Food Standards Agency (original version)
FSAm-NPS: Nutrient Profiling System of the British Food Standards Agency (modified version)
HCSP: High Council for Public Health
HR: hazard ratio
ICD-O: International Classification of Diseases for Oncology
NACRe: Network for Nutrition And Cancer Research
ONQI-f: Overall Nutritional Quality Index
WCRF: World Cancer Research Fund
