# Supplementary figures and images for: Nutritional quality of food as represented by the FSAm-NPS nutrient profiling system underlying the Nutri-Score label and cancer risk in Europe: Results from the EPIC prospective cohort study
Source: PLoS Med. 2018 Sep 18;15(9):e1002651. doi: 10.1371/journal.pmed.1002651 (PMC6143197; doi:10.1371/journal.pmed.1002651)

**S1 Fig. The Nutri-Score front-of-pack nutritional label (Santé Publique France)**

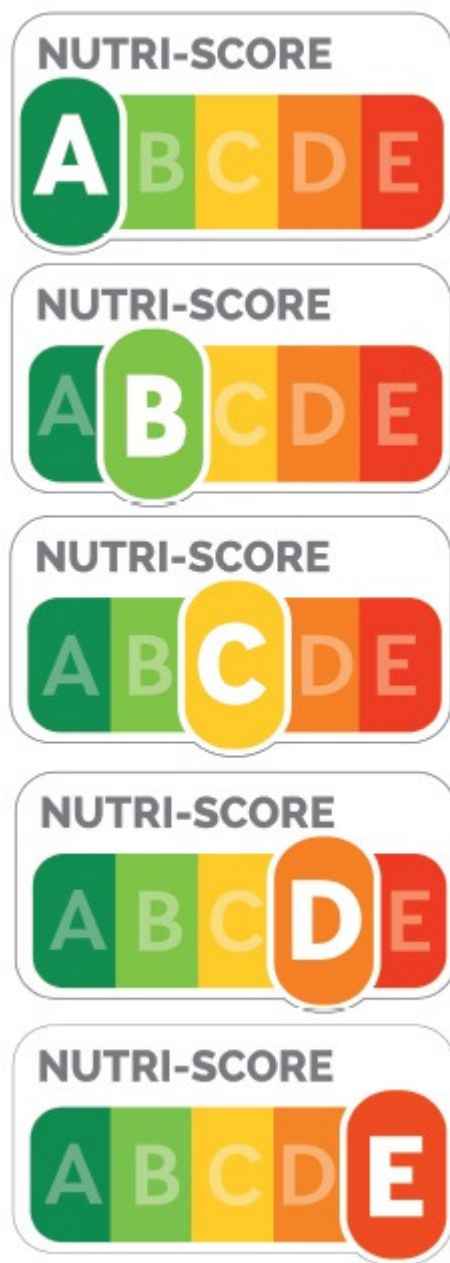

Supplement: S1 Fig — (PDF) [file pmed.1002651.s001.pdf]
