# Supplementary material for: Nutritional quality of food as represented by the FSAm-NPS nutrient profiling system underlying the Nutri-Score label and cancer risk in Europe: Results from the EPIC prospective cohort study
Source: PLoS Med. 2018 Sep 18;15(9):e1002651. doi: 10.1371/journal.pmed.1002651 (PMC6143197; doi:10.1371/journal.pmed.1002651)

S2 Fig. Participants' flowchart, EPIC cohort, 1992–2014. EPIC, European Prospective Investigation into Cancer and Nutrition.

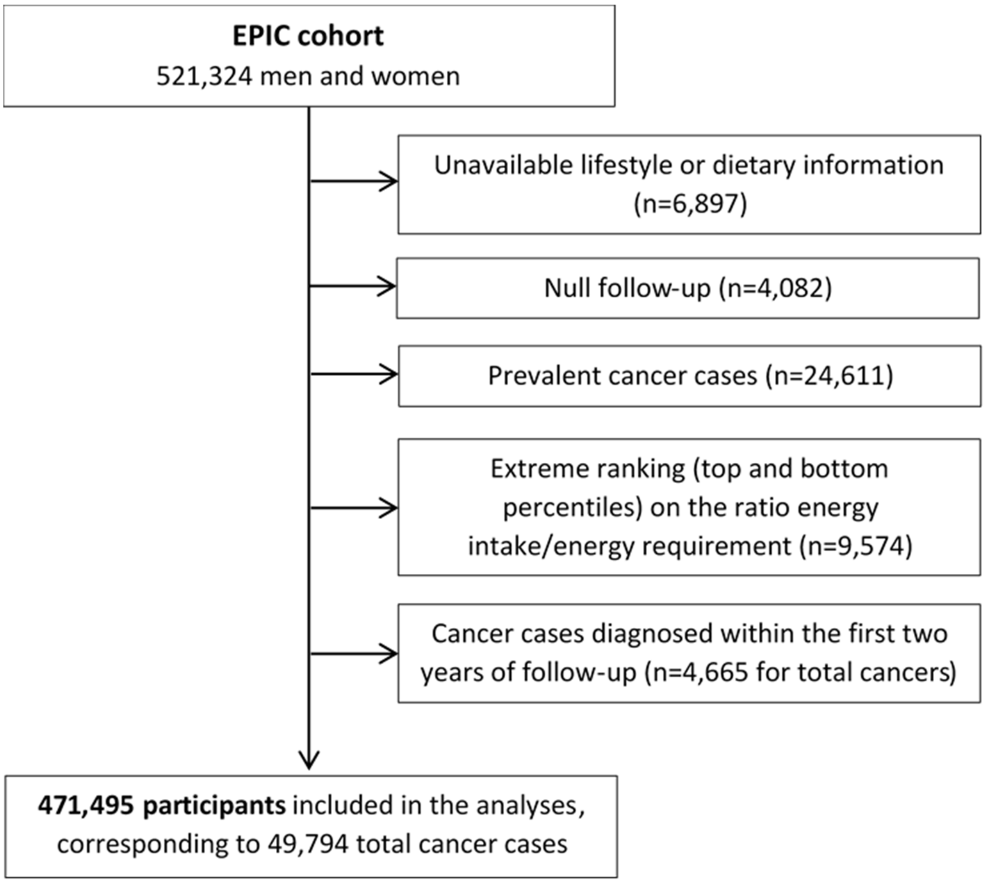

Supplement: S2 Fig — EPIC, European Prospective Investigation into Cancer and Nutrition. (PDF) [file pmed.1002651.s002.pdf]
