# Supplementary material for: Nutritional quality of food as represented by the FSAm-NPS nutrient profiling system underlying the Nutri-Score label and cancer risk in Europe: Results from the EPIC prospective cohort study
Source: PLoS Med. 2018 Sep 18;15(9):e1002651. doi: 10.1371/journal.pmed.1002651 (PMC6143197; doi:10.1371/journal.pmed.1002651)
Supplement: S1 Table — EPIC, European Prospective Investigation into Cancer and Nutrition. (PDF) [file pmed.1002651.s003.pdf]

S1 Table. Incident cancer cases and noncases by country, EPIC cohort, 1992–2014. EPIC, European Prospective Investigation into Cancer and Nutrition.

	Country									
	**France**	**Italy**	**Spain**	**United Kingdom**	**The Netherlands**	**Greece**	**Germany**	**Sweden**	**Denmark**	**Norway**
**Non-case**	62,310 (93.3%)	39,840 (90.3%)	35,822 (90.1%)	66,846 (89.5%)	32,768 (90.5%)	24,776 (95.8%)	44,298 (92.2%)	40,136 (83.5%)	43,962 (81.1%)	30,943 (91.8%)
**Incident cancer cases**	4456 (6.67%)	4285 (9.71%)	3922 (9.87%)	7859 (10.5%)	3443 (9.51%)	1092 (4.22%)	3768 (7.84%)	7942 (16.5%)	10,279 (18.9%)	2748 (8.16%)
