# Supplementary material for: Nutritional quality of food as represented by the FSAm-NPS nutrient profiling system underlying the Nutri-Score label and cancer risk in Europe: Results from the EPIC prospective cohort study
Source: PLoS Med. 2018 Sep 18;15(9):e1002651. doi: 10.1371/journal.pmed.1002651 (PMC6143197; doi:10.1371/journal.pmed.1002651)
Supplement: S2 Table — DI, Dietary Index; EPIC, European Prospective Investigation into Cancer and Nutrition; FSAm-NPS, Nutrient Profiling System of the British Food Standards Agency (modified version). (PDF) [file pmed.1002651.s004.pdf]

S2 Table. Associations between the FSAm-NPS DI and risk for cancers of the female reproductive system, by menopausal status, from multivariable Cox proportional hazards models, EPIC cohort, 1992–2014. DI, Dietary Index; EPIC, European Prospective Investigation into Cancer and Nutrition; FSAm-NPS, Nutrient Profiling System of the British Food Standards Agency.

			**FSAm-NPS DI**					
	**Per 2-point increment**	**P-trend**	**Q1**	**Q2**	**Q3**	**Q4**	**Q5**	**P-trend**
**FSAm-NPS DI range (women)**			-4.3-4.1	4.1-5.3	5.3-6.4	6.4-7.7	7.7-18.9	
**Breast cancer**								
Pre-menopause (cases/person-years)	*2512/1,603,876*		*431/315,711*	*519/320,503*	*479/326,589*	*551/317,639*	*532/323,433*	
Unadjusted model - HR (95% CI)[a]	1.00 (0.95-1.04)	0.8	1.00 (ref)	1.11 (0.97-1.26)	0.97 (0.85-1.11)	1.08 (0.94-1.24)	1.00 (0.87-1.15)	0.8
Multi-adjusted model - HR (95% CI)[b]	1.00 (0.95-1.04)	0.9	1.00 (ref)	1.11 (0.97-1.27)	0.97 (0.85-1.11)	1.08 (0.94-1.24)	1.01 (0.87-1.17)	0.9
Post-menopause (cases/person-years)	*9551/3,058,411*		*1662/645,226*	*1784/615,086*	*1924/603,814*	*2077/603,535*	*2104/590,900*	
Unadjusted model - HR (95% CI)	1.03 (1.01-1.06)	0.003	1.00 (ref)	1.03(0.97-1.11)	1.06 (0.99-1.13)	1.09 (1.02-1.17)	1.10 (1.03-1.18)	0.004
Multi-adjusted model - HR (95% CI)	1.03 (1.00-1.05)	0.03	1.00 (ref)	1.02 (0.96-1.10)	1.04 (0.98-1.12)	1.07 (1.00-1.15)	1.08 (1.00-1.16)	0.03
**Endometrial cancer[c]**								
Pre-menopause (cases/person-years)	*223/1,604,473*		*59/315,807*	*45/320,608*	*47/326,700*	*43/317,772*	*29/323,586*	
Unadjusted model - HR (95% CI)	0.95 (0.82-1.10)	0.5	1.00 (ref)	0.82 (0.55-1.21)	0.93 (0.63-1.39)	0.99 (0.65-1.52)	0.74 (0.46-1.21)	0.5
Multi-adjusted model - HR (95% CI)	0.95 (0.82-1.10)	0.5	1.00 (ref)	0.82 (0.55-1.23)	0.95 (0.63-1.42)	1.01 (0.66-1.56)	0.74 (0.44-1.22)	0.5
Post-menopause (cases/person-years)	*1540/2,928,017*		*342/611,425*	*332/586,962*	*297/578,807*	*318/580,247*	*251/570,576*	
Unadjusted model - HR (95% CI)	0.96 (0.91-1.01)	0.09	1.00 (ref)	1.03 (0.89-1.21)	0.94 (0.80-1.10)	1.03 (0.88-1.22)	0.86 (0.72-1.03)	0.2
Multi-adjusted model - HR (95% CI)	0.98 (0.93-1.04)	0.5	1.00 (ref)	1.05 (0.90-1.23)	0.98 (0.83-1.16)	1.10 (0.93-1.30)	0.94 (0.78-1.13)	0.8
**Cervical cancer[c]**								
Pre-menopause (cases/person-years)	*119/1,604,492*		*27/315,808*	*22/320,608*	*30/326,704*	*29/317,785*	*11/323,587*	
Unadjusted model - HR (95% CI)	0.92 (0.76-1.11)	0.4	1.00 (ref)	0.78 (0.44-1.38)	1.10 (0.64-1.89)	1.15 (0.65-2.01)	0.46 (0.22-0.96)	0.3
Multi-adjusted model - HR (95% CI)	0.94 (0.77-1.15)	0.5	1.00 (ref)	0.80 (0.45-1.42)	1.12 (0.65-1.93)	1.19 (0.67-2.12)	0.48 (0.22-1.04)	0.4
Post-menopause (cases/person-years)	*186/2,928,139*		*39/611,449*	*49/586,985*	*32/578,835*	*31/580,273*	*35/570,597*	
Unadjusted model - HR (95% CI)	1.12 (0.97-1.30)	0.1	1.00 (ref)	1.50 (0.98-2.30)	1.07 (0.66-1.74)	1.18 (0.71-1.95)	1.52 (0.92-2.50)	0.3
Multi-adjusted model - HR (95% CI)	1.11 (0.95-1.30)	0.2	1.00 (ref)	1.52 (0.99-2.34)	1.06 (0.65-1.73)	1.17 (0.70-1.95)	1.46 (0.86-2.46)	0.4
**Ovary cancer[c]**								
Pre-menopause (cases/person-years)	*214/1,604,460*		*36/315,807*	*40/320,604*	*51/326,687*	*39/317,779*	*48/323,583*	
Unadjusted model - HR (95% CI)	1.16 (1.01-1.33)	0.04	1.00 (ref)	1.05 (0.67-1.66)	1.39 (0.90-2.16)	1.20 (0.74-1.92)	1.57 (0.98-2.51)	0.06
Multi-adjusted model - HR (95% CI)	1.12 (0.96-1.29)	0.1	1.00 (ref)	1.05 (0.66-1.65)	1.36 (0.87-2.12)	1.14 (0.70-1.85)	1.41 (0.86-2.30)	0.2
Post-menopause (cases/person-years)	*1,059/2,928,035*		*232/611,421*	*195/586,968*	*213/578,810*	*214/580,255*	*205/570,582*	
Unadjusted model - HR (95% CI)	1.02 (0.96-1.08)	0.6	1.00 (ref)	0.90 (0.74-1.10)	1.01 (0.83-1.22)	1.03 (0.84-1.25)	1.00 (0.81-1.23)	0.6
Multi-adjusted model - HR (95% CI)	1.03 (0.96-1.10)	0.4	1.00 (ref)	0.91 (0.75-1.11)	1.03 (0.84-1.25)	1.05 (0.86-1.29)	1.03 (0.83-1.28)	0.4

[a] Unadjusted models were stratified for centre and age at recruitment (1-y intervals, time-scale)

[b] Multi-adjusted models were stratified for centre and age at recruitment (1-y intervals, time-scale) and adjusted for BMI (continuous), height (continuous), baseline alcohol intake (g/d), physical activity (Cambridge index: active, moderately active, moderately inactive, inactive, missing), smoking status and intensity of smoking (current, 1-15 cigarettes/d; current, 16-25 cigarettes/d; current, 26+ cigarettes/d; current, pipe/cigar/occasional; current/former, missing; former, quit 11-20y; former, quit 20+y; former, quit ≤ 10y; never; unknown), family history of breast cancer [breast cancer], educational level (longer education (incl. University degree), secondary school, primary school completed, not specified), baseline energy intake (kcal/d), interaction term between BMI and menopausal status, menopausal status (premenopausal, perimenopausal, postmenopausal, surgical), ever use of oral contraception (yes, no, missing), ever use of hormonal treatment for menopause (yes, no, missing), age at menarche (≤12y, 13-14y, ≥15y, missing), age at first full-term pregnancy (nulliparous, ≤21y, 22-30y, >30y, missing) and age at menopause (<50y, ≥50y).

[c] Women who declared a surgical menopause at baseline were excluded.
