# Supplementary material for: Nutritional quality of food as represented by the FSAm-NPS nutrient profiling system underlying the Nutri-Score label and cancer risk in Europe: Results from the EPIC prospective cohort study
Source: PLoS Med. 2018 Sep 18;15(9):e1002651. doi: 10.1371/journal.pmed.1002651 (PMC6143197; doi:10.1371/journal.pmed.1002651)
Supplement: S1 Text — DI, Dietary Index; FSAm-NPS, Nutrient Profiling System of the British Food Standards Agency (modified version). (PDF) [file pmed.1002651.s005.pdf]

S1 Text. FSAm-NPS score computation at food/beverage level, FSAm-NPS DI computation at individual level and link to the Nutri-Score (Santé Publique France). DI, Dietary Index; FSAm-NPS, Nutrient Profiling System of the British Food Standards Agency

1) FSAm-NPS score computation at food/beverage level

Points are allocated according to the nutrient content for 100g of foods or beverages.
Points are allocated for 'Negative' nutrients (A points) and can be balanced according to 'Positive' nutrients (C points).

A points

Total A points = (points for energy) + (points for saturated fat) + (points for total sugar) + (points for sodium)

Points	**Energy (kJ)**	**Saturated Fat (g)**	**Total Sugars (g)**	**Sodium (mg)**
0	≤ 335	≤ 1	≤ 4.5	≤ 90
1	> 335	> 1	> 4.5	> 90
2	> 670	> 2	> 9	> 180
3	> 1005	> 3	> 13.5	> 270
4	> 1340	> 4	> 18	> 360
5	> 1675	> 5	> 22.5	> 450
6	> 2010	> 6	> 27	> 540
7	> 2345	> 7	> 31	> 630
8	> 2680	> 8	> 36	> 720
9	> 3015	> 9	> 40	> 810
10	> 3350	> 10	> 45	> 900

C points

Total C points = (points for fruits/vegetables/legumes/nuts) + (points for fibres) + (points for proteins)

Points	**Fruits/vegetables/legumes/nuts (%)**	**Fibre (g) ***	**Protein (g)**
0	≤ 40	≤ 0.7	≤ 1.6
1	> 40	> 0.7	> 1.6
2	> 60	> 1.4	> 3.2
3	-	> 2.1	> 4.8
4	-	> 2.8	> 6.4
5	> 80	> 3.5	> 8.0

* FSAm-NPS score allocates different thresholds for fibres, depending on the measurement method used. We used NSP cut-offs to compute fibres score.

For 100g of a given food, the percentage of fruits/vegetables/legumes/nuts is obtained by summing up the amount (in grams) of all fruits, legumes and vegetables (including oleaginous fruits, dried fruits and olives) contained in this food.

Overall score computation

- If Total A points <11, then FSAm-NPS score =Total A points – Total C points
- If Total A points ≥11,
  - If points for fruits/vegetables/legumes/nuts =5, then FSAm-NPS score =Total A points – Total C points
  - Else if points for fruits/vegetables/legumes/nuts <5, then FSAm-NPS score = Total A points – (points for fibre + points for fruits/vegetables/legumes/nuts).

Exceptions were made for cheese, added fat, and drinks to better rank them according to their nutrient profile, consistently with nutritional recommendations:

Score computation for cheese

For cheese, the score takes in account the protein content, whether the A score reaches 11 or not, i.e.: FSAm-NPS score =Total A points – Total C points

Score computation for added fat

For added fat, the grid for point attribution is based on the percentage of saturated fat among total lipids (instead of saturated fat (g)) and has a six-point homogenous ascending step, as shown thereafter:

Points	**Saturated Fat/Lipids (%)**
0	< 10
1	< 16
2	< 22
3	< 28
4	< 34
5	< 40
6	< 46
7	< 52
8	< 58
9	< 64
10	≥ 64

Points attribution for the other nutrients follows the grid displayed in "A points" and "C points" above.

Score computation for drinks

For drinks, the grids for point attribution regarding energy, sugars and fruits/vegetables/ legumes/nuts (%) were modified.

Points	**Energy (kJ)**	**Sugars (g)**	**Fruits/vegetables/legumes/nuts (%)**
0	≤ 0	≤ 0	< 40
1	≤ 30	≤ 1.5	
2	≤ 60	≤ 3	> 40
3	≤ 90	≤ 4.5	
4	≤ 120	≤ 6	> 60
5	≤ 150	≤ 7.5	
6	≤ 180	≤ 9	
7	≤ 210	≤ 10.5	
8	≤ 240	≤ 12	
9	≤ 270	≤ 13.5	
10	> 270	> 13.5	> 80

Points attribution for the other nutrients follows the grid displayed in "A points" and "C points" above.

Given the modification of the grid for fruit and vegetables for beverages, the threshold in the final computation to take into account protein content is set at 10 points:

- If Total A points <11, then FSAm-NPS score =Total A points – Total C points
  - If Total A points ≥11,
    - If points for fruits/vegetables/legumes/nuts =10, then FSAm-NPS score =Total A points – Total C points
    - Else if points for fruits/vegetables/legumes/nuts <10, then FSAm-NPS score = Total A points – (points for fibre + points for fruits/vegetables/legumes/nuts).

Milk and vegetable milk are not concerned by this exception. Their scores are computed using the overall score computation system.

2) <u>FSAm-NPS DI computation at individual level</u>

The FSAm-NPS DI is computed at the individual level as an energy-weighted mean of the FSAm-NPS scores of all foods and beverages consumed, using the following equation [1] (FSi: score of food/beverage i, Ei: energy intake from food/beverage i, n: number of food/beverage consumed)

$$\text{FSAm-NPS DI} = \frac{\sum_{i=1}^{n}(FS_i E_i)}{\sum_{i=1}^{n} E_i}$$

Higher FSAm-NPS DI therefore reflects lower nutritional quality in foods consumed.

Energy was chosen to weight the FSAm-NPS DI over food quantity (in grams) or portion size, as previously published [1-8]. Weighting by quantity gives excessive and disproportionate weight to water and thus to beverages and foods with high water content, as tested previously [9]. Weighting by portion size was not chosen because no standardized portion sizes have been defined at the EU level and it is therefore difficult to define a reference portion size to build the score.

3) Example of FSAm-NPS score computation and link to the Nutri-Score (Santé Publique France)

Food/beverage composition

	A points				C points		
	**Energy (kJ)**	**Saturated Fat (g)**	**Total Sugars (g)**	**Sodium (mg)**	**Protein (g)**	**Fibre (g)**	**Fruits/ vegetables/ legumes/ nuts (%)**
Food							
Fennel boiled	104.7	0	0.15	73.8	1.69	2.92	100
Anchovy in vinegar	439.6	1.26	0.03	307.9	18.4	0	0
Salami	2097.6	17.5	0.13	1817.3	26.0	0	0
Beverage							
Orange juice fresh	192.6	0.02	5.50	1	0.7	0.1	100
Cola, regular	227.6	0	10.51	4.18	3.03	0	0

Attribution of points

	A points				C points		
	**Energy (kJ)**	**Saturated Fat (g)**	**Total Sugars (g)**	**Sodium (mg)**	**Protein (g)**	**Fibre (g)**	**Fruits/ vegetables/ legumes/ nuts (%)**
Food							
Fennel boiled	0	0	0	0	1	4	5
Anchovy in vinegar	1	1	0	3	5	0	0
Salami	6	10	0	10	5	0	0
Beverage							
Orange juice fresh	7	0	4	0	0	0	10
Cola, regular	8	0	8	0	1	0	0

FSAm-NPS score and Attribution of Nutri-Score colours

**Foods (points)**	**Beverages (points)**	**Colour**	
Min to -1	Water	Dark green	*Highest nutritional quality*
0 to 2	Min to 1	Light green	
3 to 10	2 to 5	Yellow	
11 to 18	6 to 9	Light orange	
19 to max	10 to max	Dark orange	*Lowest nutritional quality*

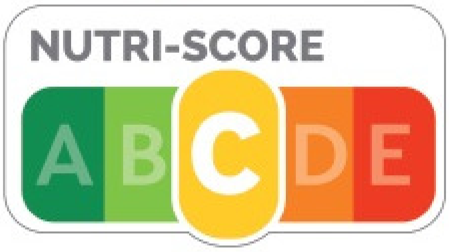

	**FSAm-NPS score**		**Nutri-Score colour**
Food			
Fennel boiled	A points – C points	**-10**	Dark green
Anchovy in vinegar	A points – C points	**0**	Light green
Salami	A points – points (fibre) – points (fruits/ veg./ leg./ nuts)	**26**	Dark orange
Beverage			
Orange juice fresh	A points – C points	**1**	Light green
Cola, regular	A points – points (fibre) – points (fruits/ veg./ leg./ nuts)	**16**	Light orange

References

(1) Julia C, Touvier M, Mejean C, Ducrot P, Peneau S, Hercberg S, et al. Development and validation of an individual dietary index based on the British Food Standard Agency nutrient profiling system in a French context. J Nutr. 2014;144:2009-17.

(2) Adriouch S, Julia C, Kesse-Guyot E, Mejean C, Ducrot P, Peneau S, et al. Prospective association between a dietary quality index based on a nutrient profiling system and cardiovascular disease risk. Eur J Prev Cardiol. 2016;23:1669-76.

(3) Adriouch S, Julia C, Kesse-Guyot E, Ducrot P, Peneau S, Mejean C, et al. Association between a dietary quality index based on the food standard agency nutrient profiling system and cardiovascular disease risk among French adults. Int J Cardiol. 2017;234:22-7.

(4) Deschasaux M, Julia C, Kesse-Guyot E, Lecuyer L, Adriouch S, Mejean C, et al. Are self-reported unhealthy food choices associated with an increased risk of breast cancer? Prospective cohort study using the British Food Standards Agency nutrient profiling system. BMJ Open. 2017;7:e013718.

(5) Donnenfeld M, Julia C, Kesse-Guyot E, Mejean C, Ducrot P, Peneau S, et al. Prospective association between cancer risk and an individual dietary index based on the British Food Standards Agency Nutrient Profiling System. Br J Nutr. 2015;114:1702-10.

(6) Julia C, Ducrot P, Lassale C, Fezeu L, Mejean C, Peneau S, et al. Prospective associations between a dietary index based on the British Food Standard Agency nutrient profiling system and 13-year weight gain in the SU.VI.MAX cohort. Prev Med. 2015;81:189-94.

(7) Julia C, Mejean C, Touvier M, Peneau S, Lassale C, Ducrot P, et al. Validation of the FSA nutrient profiling system dietary index in French adults-findings from SUVIMAX study. Eur J Nutr. 2016;55:1901-10.

(8) Julia C, Fezeu LK, Ducrot P, Mejean C, Peneau S, Touvier M, et al. The Nutrient Profile of Foods Consumed Using the British Food Standards Agency Nutrient Profiling System Is Associated with Metabolic Syndrome in the SU.VI.MAX Cohort. J Nutr. 2015;145:2355-61.

(9) Scarborough P, Arambepola C, Kaur A, Bhatnagar P, Rayner M. Should nutrient profile models be 'category specific' or 'across-the-board'? A comparison of the two systems using diets of British adults. Eur J Clin Nutr. 2010;64:553-60.
