# Supplementary material for: Nutritional quality of food as represented by the FSAm-NPS nutrient profiling system underlying the Nutri-Score label and cancer risk in Europe: Results from the EPIC prospective cohort study
Source: PLoS Med. 2018 Sep 18;15(9):e1002651. doi: 10.1371/journal.pmed.1002651 (PMC6143197; doi:10.1371/journal.pmed.1002651)
Supplement: S2 Text — (PDF) [file pmed.1002651.s006.pdf]

S2 Text. Analysis plan extracted from the project protocol submitted for funding application

Partie III : Description du projet / *Part III: Project description*

Projet scientifique / *Scientific project*

**Scientific project**
**[…]**<br>**Hypothesis and research work's main objectives**<br>The hypothesis behind this project is that a diet composed of food products with a "healthier" FSAm-NPS score is associated with a decreased risk of cancer. The largest cohort study in Europe, including dietary information as well as in depth information on disease outcomes such as cancer, is the EPIC cohort, which includes a wide and diverse European population with varied diets.<br>The main objective of this project is therefore to study the associations between the FSA-NPS DI score and cancer risk at all major anatomic sites in a diverse European population *(full EPIC cohort).*<br>**[…]**<br>**Analysis plan: FSA-NPS DI and cancer risk in the full EPIC cohort**<br>The FSA-NPS DI associated to the usual dietary intakes assessed at baseline will be prospectively analysed in relation to the risk of cancer overall and considering the main cancer locations in the full EPIC cohort. Hazard ratios and 95% confidence intervals will be estimated using Cox proportional hazards regression models with age as the primary time variable. Analyses will be stratified by age at recruitment (1-year interval) and study centre. Stratification by sex will also be considered for analyses including all non-sex-specific cancer locations (e.g. colon-rectum, bladder, lung). The following potential confounding factors, which are known risk factors for cancer and are usually associated with the nutritional quality of the diet will be considered to adjust the models, depending on the studied cancer location: anthropometric factors (e.g. BMI, height, waist circumference, waist-to-hip ratio), physical activity, smoking status, educational level, family history of cancer in first degree relatives, alcohol intake, age at menarche, menopausal status, age at menopause, use and duration of hormonal treatment for menopause, use and duration of oral contraceptives and age at birth of first child. Adjustment for dietary energy intakes will be tested, although energy intake is already included in the FSA-NPS DI computation. Participants will contribute to the Cox model until their date of cancer diagnosis, their date of death, their date of emigration/loss to follow-up or the closure date, whichever occurred first. The FSA-NPS DI will be studied as a continuous variable and as a categorical variable (e.g. sex-specific quintiles) to compare participants with the highest FSA-NPS DI scores (lowest nutritional quality of diet) to participants with the lowest scores. Percentages of increase/decrease in cancer risk across the different levels of the FSA-NPS DI will be computed.<br>Analyses will be performed using SAS version 9.4 (SAS Institute).<br>**[…]**
