# Supplementary material for: Nutritional quality of food as represented by the FSAm-NPS nutrient profiling system underlying the Nutri-Score label and cancer risk in Europe: Results from the EPIC prospective cohort study
Source: PLoS Med. 2018 Sep 18;15(9):e1002651. doi: 10.1371/journal.pmed.1002651 (PMC6143197; doi:10.1371/journal.pmed.1002651)
Supplement: S1 STROBE Statement — (PDF) [file pmed.1002651.s007.pdf]

S1 STROBE Statement—checklist of items that should be included in reports of observational studies

	Item No	Recommendation	Page
**Title and abstract**	1	(*a*) Indicate the study's design with a commonly used term in the title or the abstract	Title; Abstract: Methods and Findings
		(*b*) Provide in the abstract an informative and balanced summary of what was done and what was found	Abstract: Methods and Findings
**Introduction**			
Background/rationale	2	Explain the scientific background and rationale for the investigation being reported	Introduction §1-3
Objectives	3	State specific objectives, including any prespecified hypotheses	Introduction §4
**Methods**			
Study design	4	Present key elements of study design early in the paper	Methods: Study population
Setting	5	Describe the setting, locations, and relevant dates, including periods of recruitment, exposure, follow-up, and data collection	Methods: Study population, Baseline data collection, Dietary intake assessment, FSAm-NPS DI computation, Follow-up for cancer incidence and vital status; S1 Text
Participants	6	(*a*) *Cohort study*—Give the eligibility criteria, and the sources and methods of selection of participants. Describe methods of follow-up<br>*Case-control study*—Give the eligibility criteria, and the sources and methods of case ascertainment and control selection. Give the rationale for the choice of cases and controls<br>*Cross-sectional study*—Give the eligibility criteria, and the sources and methods of selection of participants	Methods: Study population, Follow-up for cancer incidence and vital status §1; S2 Fig
		(*b*) *Cohort study*—For matched studies, give matching criteria and number of exposed and unexposed<br>*Case-control study*—For matched studies, give matching criteria and the number of controls per case	n.a
Variables	7	Clearly define all outcomes, exposures, predictors, potential confounders, and effect modifiers. Give diagnostic criteria, if applicable	Methods: FSAm-NPS DI computation, Follow-up for cancer incidence and vital status, Statistical analyses; S1 Text
Data sources/ measurement	8*	For each variable of interest, give sources of data and details of methods of assessment (measurement). Describe comparability of assessment methods if there is more than one group	Methods: Study population, Baseline data collection, Dietary intake assessment, Follow-up for cancer incidence and vital status
Bias	9	Describe any efforts to address potential sources of bias	Methods: Study population §2, Statistical

			analyses §1-3
Study size	10	Explain how the study size was arrived at	Methods: Study population §2; S2 Fig
Quantitative variables	11	Explain how quantitative variables were handled in the analyses. If applicable, describe which groupings were chosen and why	Methods: Statistical analyses §1-2; Table 1; Footnotes to Tables 2-3 and S2 Table
Statistical methods	12	(*a*) Describe all statistical methods, including those used to control for confounding	Methods: Statistical analyses
		(*b*) Describe any methods used to examine subgroups and interactions	Methods: Statistical analyses §2-3
		(*c*) Explain how missing data were addressed	Methods: Statistical analyses §2
		(*d*) *Cohort study*—If applicable, explain how loss to follow-up was addressed<br>*Case-control study*—If applicable, explain how matching of cases and controls was addressed<br>*Cross-sectional study*—If applicable, describe analytical methods taking account of sampling strategy	Methods: Statistical analyses §1
		(*e*) Describe any sensitivity analyses	Methods: Statistical analyses §2-3
**Results**			
Participants	13*	(a) Report numbers of individuals at each stage of study—eg numbers potentially eligible, examined for eligibility, confirmed eligible, included in the study, completing follow-up, and analysed	Methods: Study population §2; S2 Fig
		(b) Give reasons for non-participation at each stage	Methods: Study population §2; S2 Fig
		(c) Consider use of a flow diagram	S2 Fig
Descriptive data	14*	(a) Give characteristics of study participants (eg demographic, clinical, social) and information on exposures and potential confounders	Results §2; Table 1
		(b) Indicate number of participants with missing data for each variable of interest	Table 1
		(c) *Cohort study*—Summarise follow-up time (eg, average and total amount)	Results §1; Tables 2-3; S2 Table
Outcome data	15*	*Cohort study*—Report numbers of outcome events or summary measures over time	Results §1; Tables 2-3; S1-2 Tables
		Case-control study—Report numbers in each exposure category, or summary measures of exposure	/
		Cross-sectional study—Report numbers of outcome events or summary measures	/
Main results	16	(*a*) Give unadjusted estimates and, if applicable, confounder-adjusted estimates and their precision (eg, 95% confidence interval). Make clear which confounders were adjusted for and why they were included	Results §3-6; Tables 2-3; S2 Table; Methods: Statistical analyses §2
		(*b*) Report category boundaries when continuous variables were categorized	Tables 2-3; S2 Table
		(*c*) If relevant, consider translating estimates of relative	Results §3

		risk into absolute risk for a meaningful time period	
Other analyses	17	Report other analyses done—eg analyses of subgroups and interactions, and sensitivity analyses	Results §6
**Discussion**			
Key results	18	Summarise key results with reference to study objectives	Discussion §1
Limitations	19	Discuss limitations of the study, taking into account sources of potential bias or imprecision. Discuss both direction and magnitude of any potential bias	Discussion §7-8
Interpretation	20	Give a cautious overall interpretation of results considering objectives, limitations, multiplicity of analyses, results from similar studies, and other relevant evidence	Discussion §2-9
Generalisability	21	Discuss the generalisability (external validity) of the study results	Discussion §2-8
**Other information**			
Funding	22	Give the source of funding and the role of the funders for the present study and, if applicable, for the original study on which the present article is based	Funding

*Give information separately for cases and controls in case-control studies and, if applicable, for exposed and unexposed groups in cohort and cross-sectional studies.

Note: An Explanation and Elaboration article discusses each checklist item and gives methodological background and published examples of transparent reporting. The STROBE checklist is best used in conjunction with this article (freely available on the Web sites of PLoS Medicine at http://www.plosmedicine.org/, Annals of Internal Medicine at http://www.annals.org/, and Epidemiology at http://www.epidem.com/). Information on the STROBE Initiative is available at www.strobe-statement.org.
